# Tracking of progressing human DNA polymerase δ holoenzymes reveals distributions of DNA lesion bypass activities

**DOI:** 10.1093/nar/gkac745

**Published:** 2022-09-15

**Authors:** Rachel L Dannenberg, Joseph A Cardina, Kara G Pytko, Mark Hedglin

**Affiliations:** Department of Chemistry, The Pennsylvania State University, University Park, PA 16802, USA; Department of Chemistry, The Pennsylvania State University, University Park, PA 16802, USA; Department of Chemistry, The Pennsylvania State University, University Park, PA 16802, USA; Department of Chemistry, The Pennsylvania State University, University Park, PA 16802, USA

## Abstract

During DNA replication, DNA lesions in lagging strand templates are initially encountered by DNA polymerase δ (pol δ) holoenzymes comprised of pol δ and the PCNA processivity sliding clamp. These encounters are thought to stall replication of an afflicted template before the lesion, activating DNA damage tolerance (DDT) pathways that replicate the lesion and adjacent DNA sequence, allowing pol δ to resume. However, qualitative studies observed that human pol δ can replicate various DNA lesions, albeit with unknown proficiencies, which raises issues regarding the role of DDT in replicating DNA lesions. To address these issues, we re-constituted human lagging strand replication to quantitatively characterize initial encounters of pol δ holoenzymes with DNA lesions. The results indicate pol δ holoenzymes support dNTP incorporation opposite and beyond multiple lesions and the extent of these activities depends on the lesion and pol δ proofreading. Furthermore, after encountering a given DNA lesion, subsequent dissociation of pol δ is distributed around the lesion and a portion does not dissociate. The distributions of these events are dependent on the lesion and pol δ proofreading. Collectively, these results reveal complexity and heterogeneity in the replication of lagging strand DNA lesions, significantly advancing our understanding of human DDT.

## INTRODUCTION

**Figure 1.**
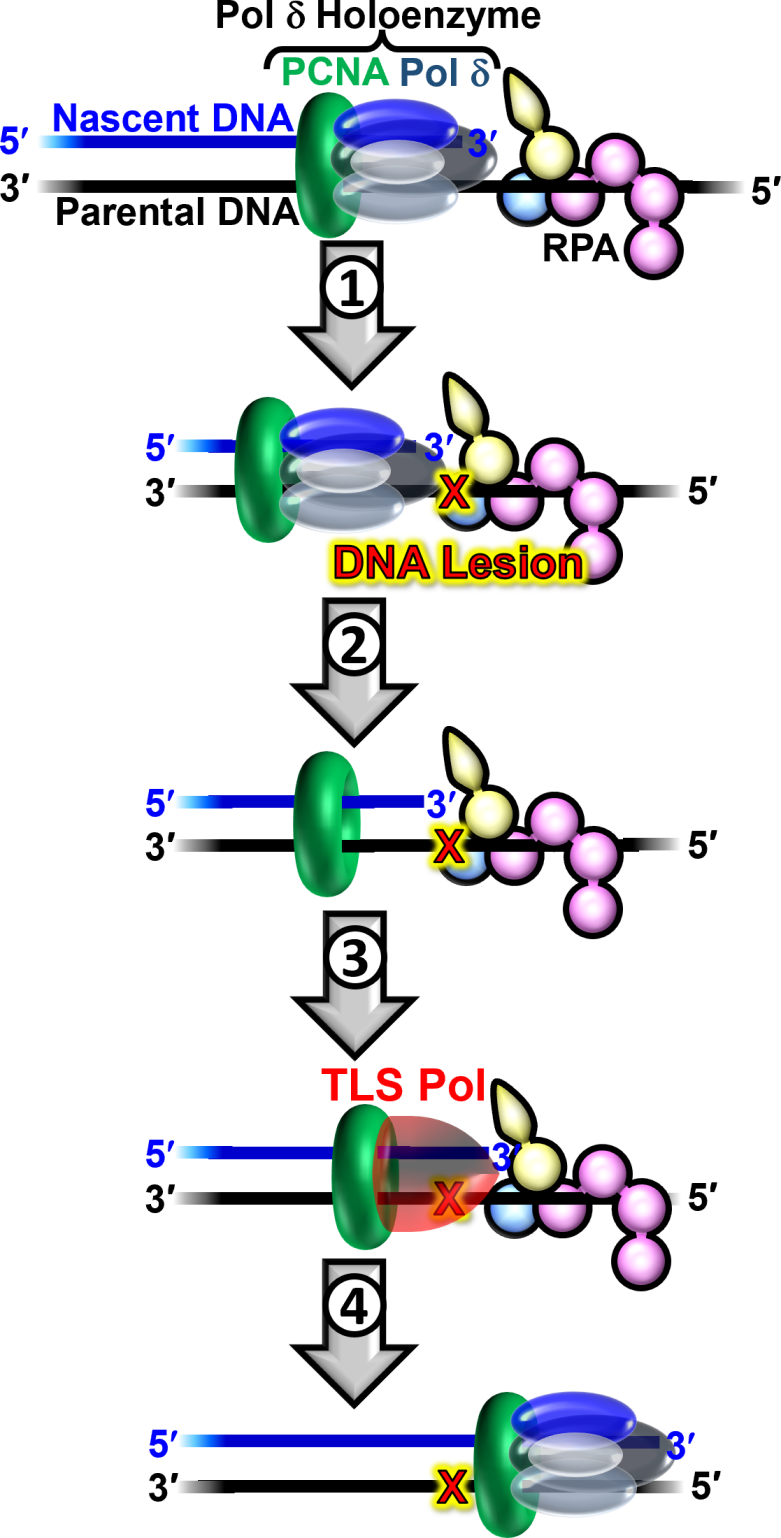
DNA damage tolerance in lagging strand templates. At the top, a progressing pol δ holoenzyme (pol δ + PCNA) is depicted replicating a lagging strand template engaged by RPA. **1**) A progressing pol δ holoenzyme encounters a DNA lesion in a lagging strand template. **2**) Pol δ rapidly and passively dissociates into solution, leaving PCNA and RPA behind on the DNA. Pol δ may reiteratively associate and dissociate to/from the resident PCNA encircling the stalled P/T junction but cannot support stable dNTP incorporation opposite the offending DNA lesion. **3**) The stalled P/T junction activates one or more DNA damage tolerance pathway(s) that are ultimately responsible for the insertion of a dNTP opposite the lesion (insertion), extension of the nascent DNA 1 nt downstream of the lesion (extension), and possibly further extension of the nascent DNA > 1 nt downstream of the lesion (elongation). TLS is depicted as an example of DDT. In this pathway, one or more TLS polymerases engage PCNA encircling the aborted P/T junction and perform insertion, extension, and (possibly) elongation. **4**) After DDT is complete, replication by a pol δ holoenzyme may resume downstream of the lesion. In this view, DDT is solely responsible for the replication of a DNA lesion (i.e. insertion), and, hence, pol δ does not contribute to the fidelity of replicating DNA lesions.

In humans, like all eukaryotes, lagging strand DNA templates are primarily replicated by DNA polymerase δ (pol δ, Figure 1, top), which is a member of the B-family of DNA polymerases. Pol δ is comprised of four subunits; three accessory subunits (p50/POLD2, p66/POLD3 and p12/POLD4) and a catalytic subunit (p125/POLD1) that contains distinct active sites for DNA polymerase and 3′ → 5′ exonuclease (i.e. proofreading) activities (1). On its own, human pol δ is an inefficient and distributive DNA polymerase and must anchor to the processivity sliding clamp, proliferating cell nuclear antigen (PCNA), to form a pol δ holoenzyme with maximal efficiency and processivity (2). The highly conserved ring-shaped structure of PCNA has a central cavity large enough to encircle double-stranded DNA (dsDNA) and slide freely along it (3). Thus, association of pol δ with PCNA encircling a primer/template (P/T) junction effectively tethers the polymerase to DNA, substantially increasing the extent of continuous replication. The major single-strand DNA (ssDNA)-binding protein, replication protein A (RPA), engages the downstream template ssDNA that is to be replicated, preventing its degradation by cellular nucleases and formation of secondary DNA substrates that are prohibitive to DNA replication (4). Furthermore, upon dissociation of pol δ from a P/T junction, RPA prevents diffusion of PCNA along the adjacent 5′ ssDNA overhang (5,6).

**Figure 2.**
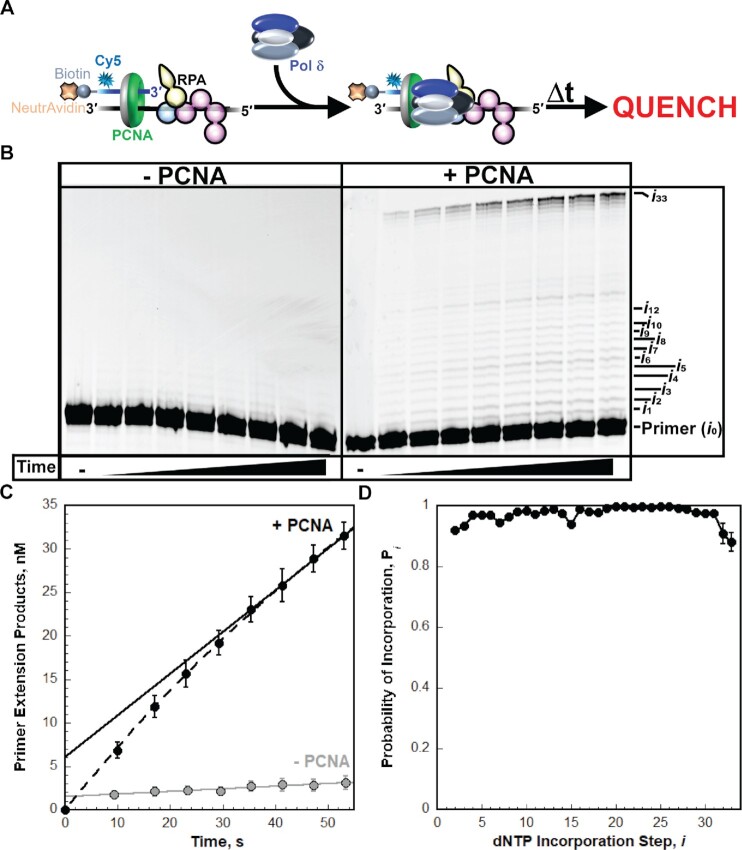
Replication by pol δ holoenzymes. (**A**) Schematic representation of the experiment performed to monitor primer extension by pol δ holoenzymes during a single binding encounter with a P/T DNA substrate. (**B**) 16% denaturing sequencing gel of the primer extension products observed for the native (i.e. undamaged) DNA substrate (BioCy5P/T, Supplementary Figure S1). The incorporation step (*i*) for certain primer extension products (*i_1_* to *i*_10_, *i*_12_ and *i*_33_) is indicated on the far right. Shown on the left and the right are representative gels of primer extension by pol δ observed in the absence (‘–PCNA’) and presence (‘+ PCNA’) of PCNA, respectively. (**C**) Quantification of the (total) primer extension products. Data is plotted as a function of time (after the addition of pol δ) and display ‘burst’ kinetics. Data points within the ‘linear’ phase are fit to a linear regression where the Y-intercept (in nM) represents the amplitude of the ‘burst’ phase, and the slope represents the initial velocity (in nM/s) of the linear phase. Data for experiments carried out in the absence (‘–PCNA’) and presence (‘+PCNA’) of PCNA are displayed in grey and black, respectively. Data for experiments carried out in the presence of PCNA are fit to a burst + linear phase kinetic model (dashed line) only for visualizing the conformity of the linear phases for each fit. (**D**) Processivity of pol δ holoenzymes. The probability of incorporation (*P*_i_) for each dNTP incorporation step (*i*) beyond the first incorporation step is calculated as described in *Materials and Methods*. *P*_*i*_ values observed in the presence of PCNA are plotted as a function of the dNTP incorporation step *i*. Data is fit to an interpolation only for observation.

As the primary lagging strand DNA polymerase, pol δ is the first to encounter lagging strand template nucleotides that have been damaged by covalent modifications. These damaging modifications, referred to as DNA lesions, arise from exposure of genomic DNA to reactive metabolites and environmental mutagens. Given the highly stringent DNA polymerase activity of human pol δ along with its robust, intrinsic proofreading activity, the historical view for what transpires upon human pol δ encountering a DNA lesion (Figure 1, step 1) is that pol δ dissociates into solution, leaving PCNA and RPA behind at the aborted P/T junction (Figure 1, step 2). Pol δ may re-iteratively associate and dissociate from the resident PCNA, but it cannot support stable insertion of a dNTP opposite the lesion. Consequently, replication of the lagging strand template stalls, activating DNA damage tolerance (DDT) pathways (Figure 1, step 3) that are ultimately responsible for insertion of a dNTP opposite the lesion (i.e. insertion), extension of the nascent DNA 1 nucleotide (nt) downstream of the lesion (i.e. extension), and possibly further elongation of the nascent DNA >1 nt downstream of the lesion (i.e. elongation). For example, in translesion DNA synthesis (TLS), the predominant human DDT pathway, one or more TLS DNA polymerases engage PCNA encircling an aborted P/T junction and replicate the offending DNA damage and downstream template nucleotide(s) with high proficiency due to their expanded DNA polymerase active sites and lack of associated proofreading activities. After DDT, replication by pol δ holoenzymes may resume downstream of the lesion (Figure 2, step 4) (7,8).

Over the last 15 years or so, numerous qualitative studies from independent groups (9–15) observed that human pol δ is capable of replicating various DNA lesions, which raised an issue of whether pol δ is directly involved in DDT and hence a major player in the fidelity of replicating DNA lesions. This issue has critical implications for our understanding of when, how, and why DDT is activated in humans but remained unresolved as the proficiencies of DNA lesion bypass by human pol δ holoenzymes have yet to be determined. For example, if human pol δ holoenzymes are proficient at insertion for a given DNA lesion, then DDT may only be activated to perform extension and possibly elongation. To address this issue, we re-constituted human lagging strand replication at physiological pH, ionic strength, and dNTP concentrations to quantitatively characterize, at single nucleotide resolution, the initial encounters of pol δ holoenzymes with downstream DNA lesions. In short, a DNA lesion ≥9 nt downstream of a P/T junction is encountered only once and only by a progressing pol δ holoenzyme, rather than pol δ alone. To the best of our knowledge, comparable studies of human lagging strand replication have yet to be reported. The results indicate that human pol δ holoenzymes support stable dNTP incorporation opposite and beyond multiple lesions and the extent of these activities depends on the identity of the lesion and the ability to proofread intrinsically (as opposed to extrinsically). Furthermore, the results indicate that, after encountering a given DNA lesion, subsequent dissociation of pol δ does not conform to a uniform site relative to the lesion. Rather, pol δ dissociation events are distributed around the lesion and a portion of pol δ does not dissociate at all. The distributions of these events are dependent on the identity of the lesion and the ability to proofread intrinsically. The results from the present study together with those from previous reports on human pol δ reveal complexity and heterogeneity in the replication of lagging strand DNA lesions, significantly advancing our understanding of human DDT.

## MATERIALS AND METHODS

### Recombinant human proteins

Human RPA, Cy5-PCNA, RFC and pol δ (exonuclease-deficient and wild-type) were obtained as previously described (16,17). The concentration of active RPA was determined via a FRET-based activity assay as described previously (18).

### Oligonucleotides

Oligonucleotides were synthesized by Integrated DNA Technologies (Coralville, IA) or Bio-Synthesis (Lewisville, TX) and purified on denaturing polyacrylamide gels. For oligonucleotides containing a thymine glycol, the *cis*-5*R*,6*S* stereoisomer of the DNA lesion is incorporated into the oligonucleotide sequence (confirmed by Bio-synthesis). This stereoisomer of Tg is the most abundant and the most stable (19). The concentrations of unlabeled DNAs were determined from the absorbance at 260 nm using the calculated extinction coefficients. The concentrations of Cy5-labeled DNAs were determined from the extinction coefficient at 650 nm for Cy5 (ϵ_650_ = 250 000 M^−1^cm^−1^). The concentrations of Cy3-labeled DNAs were determined from the extinction coefficient at 550 nm for Cy3 (ϵ_650_ = 125 000 M^−1^ cm^−1^). For annealing two single strand DNAs, the primer and corresponding, complementary template strand were mixed in equimolar amounts in 1× Annealing Buffer (10 mM Tris–HCl, pH 8.0, 100 mM NaCl, 1 mM EDTA), heated to 95°C for 5 min, and allowed to slowly cool to room temperature.

### Primer extension assays

All primer extension experiments were performed at 25°C in an assay buffer consisting of 1× Replication Buffer supplemented with 1 mM DTT and 1 mM ATP. For all experiments, the final ionic strength was adjusted to 230 mM by addition of appropriate amounts of KOAc and samples are protected from light whenever possible. All reagents, substrate, and protein concentrations listed are final reaction concentrations. First, 250 nM of a Cy5-labeled P/T DNA (Supplementary Figure S1) is preincubated with 1 μM Neutravidin. Next, RPA (750 nM heterotrimer) is added and the resultant mixture is allowed to equilibrate for 5 min. PCNA (250 nM homotrimer), ATP (1 mM), and RFC (250 nM heteropentamer) are then added in succession and the resultant mixture is incubated for 5 min. Finally, dNTPs (46 μM dATP, 9.7 μM dGTP, 48 μM dCTP, 67 μM dTTP) are added and DNA synthesis is initiated by the addition of limiting pol δ (either 8.8 nM wild-type or 35 nM exonuclease-deficient heterotetramer). The concentration of each dNTP utilized is within the physiological range observed in dividing human cells (24 ± 22 μM dATP, 5.2 ± 4.5 μM dGTP, 29 ± 19 μM dCTP, 37 ± 30 μM dTTP) (20). At variable times, aliquots of the reaction were removed, quenched with 62.5 mM EDTA, pH 8.0, 2 M urea, 50% formamide supplemented with 0.01% (wt/vol) tracking dyes. In general, primer extension assays utilizing wild-type pol δ were monitored for ≤60 s and those utilizing exonuclease-deficient pol δ were monitored for ≤120 s. Primer extension products were resolved on 16% sequencing gels. Before loading onto a gel, quenched samples were heated at 95°C for 5 min and immediately chilled in ice water for 5 min. Gel images were obtained on a Typhoon Model 9410 imager. The fluorescence intensity in each band on a gel was quantified with ImageQuant (GE Healthcare) and the fluorescence intensity of each DNA band within a given lane was converted to concentration by first dividing its intensity by the sum of the intensities for all of the species present in the respective lane and then multiplying the resultant fraction by the concentration of P/T DNA (250 nM). Within a given lane, the probability of incorporation, *P*_i_, for each dNTP incorporation step, *i*, after *i*_1_ was calculated as described previously (2,21). The insertion probability is the probability of dNTP incorporation opposite a DNA lesion or the corresponding native nucleotide at dNTP incorporation step *i* and is equal to *P*_i_. The insertion efficiency was calculated by dividing the insertion probability for a given DNA lesion by the insertion probability for the corresponding native nucleotide in the same sequence context and then multiplying the resultant quotient by 100%. The extension probability is the probability of dNTP incorporation 1 nt downstream of a DNA lesion or the corresponding native nucleotide at dNTP incorporation step *i* and is equal to *P*_*i*+1_. The extension efficiency was calculated by dividing the extension probability for a given DNA lesion by the extension probability for the corresponding native nucleotide in the same sequence context and then multiplying the resultant quotient by 100%. The bypass probability for a given DNA lesion or the corresponding native nucleotide at dNTP insertion step, *i*, was calculated by multiplying *P*_i_ by *P*_*i*+1._ The bypass probability represents the probability of dNTP incorporation opposite a DNA lesion or the corresponding native nucleotide at dNTP incorporation step *i*, and the next dNTP incorporation step downstream (*i* + 1). The bypass efficiency was calculated by dividing the bypass probability for a given DNA lesion by the bypass probability for the corresponding native nucleotide in the same sequence context and then multiplying the resultant quotient by 100%. Upon encountering a given DNA lesion or the corresponding native nucleotide at dNTP incorporation step, *i*, the fraction of pol δ that subsequently dissociates at dNTP incorporation step *i* or any dNTP incorporation step downstream is defined as the band intensity at that dNTP incorporation step divided by the sum of the band intensity at that dNTP incorporation step and the band intensities for all longer primer extension products. These values are utilized to determine the distribution (%) of pol δ dissociation events that occur after the initial encounter with a DNA lesion or the corresponding native nucleotide at dNTP incorporation step *i*. Only data points that are <20% of the reaction progress (based on the accumulation of primer extension products) are displayed in the gel images, plotted as a function of time, and analyzed with Kaleidagraph (Synergy). Within the linear phase of primer extension, the *P*_*i*_ values, the variables calculated from *P*_*i*_ values, and all parameters discussed above remain constant with incubation time. For a given dNTP incorporation step, the *P*_i_ values within the linear phase of primer extension, the variables calculated from these *P*_*i*_ values, and all parameters discussed above are each fit to a flat line where the *y*-intercept reflects the average value. For all plots in all figures, each data point/column represents the average ± S.E.M. of at least three independent experiments. Error bars are present for all data points on all plots in all figures but may be smaller than the data point.

## RESULTS

### Strategy to monitor progression of human pol δ holoenzymes

The approach utilizes P/T DNA substrates (Supplementary Figure S1) that mimic nascent P/T junctions on a lagging strand. Each P/T DNA is comprised of a 62-mer template strand annealed to a 29-mer primer strand that contains a biotin at the 5′ terminus and an internal Cy5 dye label 4 nt from the 5′ terminus. When pre-bound to Neutravidin, the biotin prevents loaded PCNA from sliding off the dsDNA end of the substrate. The lengths (29 base pairs, bp) of the dsDNA regions are identical and in agreement with the requirements for assembly of a single PCNA ring onto DNA by RFC (5,6,17). The lengths (33 nt) of the ssDNA regions adjacent to the 3′ end of the P/T junctions are identical and accommodate 1 RPA molecule (22–24). P/T DNA is pre-saturated with Neutravidin and RPA and then PCNA is assembled onto all P/T junctions by RFC and stabilized by RPA and Neutravidin/biotin blocks that prohibit PCNA from diffusing off the P/T DNA (Supplementary Figure S2) (5,6). Finally, primer extension (i.e. dNTP incorporation) is initiated by the addition of limiting pol δ (Figure 2A) and Cy5-labeled DNA products are resolved on a denaturing polyacrylamide gel (Figure 2B), visualized on a fluorescent imager, and quantified.

Under the conditions of the assay (physiological pH, ionic strength, and concentration of each dNTP), primer extension on a control (i.e. native/undamaged) P/T DNA (BioCy5P/T, Supplementary Figure S1) is severely limited in the absence of PCNA and not observed beyond the fifth dNTP incorporation step (i.e. *i*_5_) (Figure 2B, ‘–PCNA’) whereas significant primer extension is observed in the presence of PCNA up to and including the last dNTP incorporation step (*i*_33_) (Figure 2B, ‘+PCNA’). In the absence of PCNA, only 1.249 ± 0.314% of the primer is extended over the time period monitored (60 s) compared to 12.60 ± 0.61% in the presence of PCNA (Figure 2C). These observations agree with the inability of pol δ alone to form a stable complex with native P/T DNA (2,25). Altogether, the results from Figure 2A–C indicate that nearly all DNA synthesis (> 90%) observed in the presence of PCNA is carried out by pol δ holoenzymes and only pol δ holoenzymes are responsible for primer extension beyond the fifth dNTP incorporation step (i.e. *i* ≥ 5).

All primer extension assays reported in this study were performed in the presence of a large excess of P/T DNA over pol δ and only monitor ≤20% of the reaction such that once a primer is extended and the associated pol δ subsequently disengages, the probability that the extended primer will be utilized again is negligible. Rather, the dissociated pol δ engages another, previously unused primer. In other words, the observed primer extension products reflect a single cycle (i.e. single pass, single hit, etc.) of DNA synthesis. Appropriate single hit conditions are operating for any pol δ:P/T DNA ratio when the probabilities of dNTP incorporation (*P*_*i*_) remain constant with incubation time, as depicted in Supplementary Figure S3 for the BioCy5P/T DNA substrate in the presence of PCNA (2,21,26,27). This condition is met for all *P*_*i*_ values reported in this study. For a given dNTP incorporation step *i*, the probability of dNTP incorporation, *P*_i_, represents the likelihood that pol δ will incorporate a dNTP rather than dissociate. For the BioCy5P/T DNA substrate, the *P*_i_ values observed in the presence of PCNA (Figure 2D) are high and range from 0.998 ± 0.001 for the 23rd dNTP incorporation (*i*_23_) to 0.881 ± 0.031 for the last dNTP incorporation step (*i* = 33). Maximal *P*_i_ values (≥0.990) are observed beginning at *i*_16_ and are maintained until *i*_27_, after which *P*_i_ drops off, particularly at *i*_32_ and *i*_33_, as progressing pol δ holoenzymes dissociate due to the severely diminished length (2 nt) of the single strand template. Importantly, the distribution and range of observed *P*_i_ values are in excellent agreement with values reported in previous studies on the same P/T DNA substrate where only DNA synthesis by pol δ holoenzymes is observed (2,21). This re-affirms that >90%, if not all, DNA synthesis observed in the presence of PCNA is carried out by pol δ holoenzymes, as opposed to pol δ alone. All *P*_*i*_ values observed for the BioCy5P/T DNA substrate in the presence of PCNA are less than 1.0 (Figure 2D) indicating that a proportion of progressing pol δ holoenzymes dissociate at each successive dNTP incorporation step. This behavior of pol δ holoenzymes on the native P/T DNA agrees very well with the extensively documented behavior of human pol δ holoenzymes (2,21,28–34). This assay was utilized in the present study to directly compare the progression of human pol δ holoenzymes on the native/undamaged BioCy5P/T DNA substrate (Supplementary Figure S1) to that observed on damaged P/T DNA substrates that are identical to the BioCy5P/T DNA except that a single nt ≥ 9 nt downstream of the P/T junction is altered by a chemical modification(s), i.e. DNA lesion(s) (Supplementary Figure S1). The DNA lesions examined in the present study are prominent in cells exposed to oxidizing or alkylating agents. In this ‘running start’ setup, dNTP incorporation initiates upstream of a DNA lesion and, hence, the DNA lesion is encountered by progressing pol δ holoenzymes that have a ‘running start.’ Stable assembly (i.e. loading) of PCNA onto a P/T junction is not affected by any of the DNA lesions examined in the present study (Supplementary Figure S2). Thus, any observed effect on the DNA synthesis activity of assembled pol δ holoenzymes is not attributable to the amount of PCNA loaded onto a P/T DNA junction with a DNA lesion ≥9 nt downstream.

### The effect of oxidative DNA lesions on the progression of human pol δ holoenzymes

**Figure 3.**
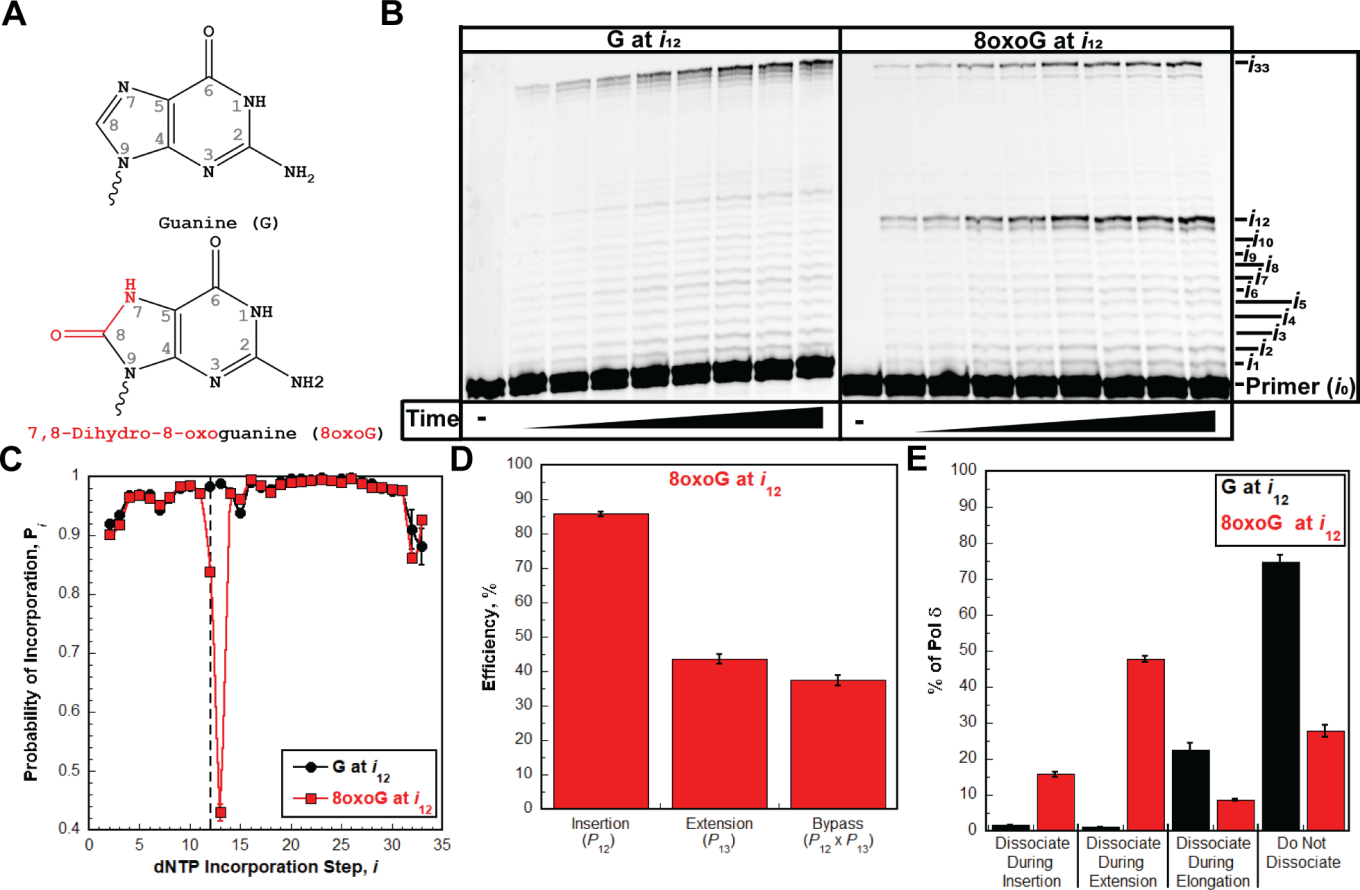
Pol δ holoenzymes encountering an 8oxoG lesion downstream of a P/T junction. The progression of human pol δ holoenzymes was monitored on a P/T DNA substrate (Bio-Cy5-P/T-8oxoG, Supplementary Figure S1) that contains an 8oxoG 12 nt downstream of the P/T junction (at the 12th dNTP incorporation step, *i*_12_). (**A**) Structure of 8oxoG. 8oxoG (Bottom) is generated from G (top) through the introduction of an oxo group on the carbon at position 8 and the addition of a hydrogen to the nitrogen at position 7. These modifications are highlighted in red on the structure of 8oxoG. (**B**) 16% denaturing sequencing gel of the primer extension products. Shown on the left and the right are representative gels of primer extension by pol δ holoenzymes on the native Bio-Cy5-P/T (‘G at *i*_12_’) and the Bio-Cy5-P/T-8oxoG (‘8oxoG at *i*_12_’) DNA substrates, respectively. (**C**) Processivity of pol δ holoenzymes. *P*_i_ values observed for the native Bio-Cy5-P/T (‘G at *i*_12_’) and the Bio-Cy5-P/T-8oxoG (‘8oxoG at *i*_12_’) DNA substrates are shown in black and red, respectively, and plotted as a function of the dNTP incorporation step *i*. Data is fit to an interpolation only for observation. Dashed line indicates dNTP incorporation step for insertion (*i*_12_). (**D**) Efficiency of replicating 8oxoG. The efficiencies for insertion, extension, and bypass are calculated as described in *Materials and Methods* and plotted as percentages. The *P*_*i*_ value(s) from which each efficiency is derived from is indicated below the respective efficiency. Values for each parameter are also reported in Supplementary Table S1. (**E**). Dissociation of pol δ holoenzymes after encountering an 8oxoG lesion at *i*_12_. The distribution of pol δ dissociation events observed for the native Bio-Cy5-P/T (‘G at *i*_12_’) and the Bio-Cy5-P/T-8oxoG (‘8oxoG at *i*_12_’) DNA substrates are indicated in black and red, respectively.

First, we examined the effect of 7,8-dihydro-8-oxoguanine (8oxoG, Figure 3A) on the progression of human pol δ holoenzymes. Guanine is the most oxidized nucleobase and 8oxoG is one of the most abundant DNA lesions generated by exposure of genomic DNA to reactive oxygen species (ROS) (35–38). The P/T DNA substrate (Bio-Cy5-P/T-8oxoG, Supplementary Figure S1) contains an 8oxoG 12 nt downstream of the P/T junction (at the 12th dNTP incorporation step, *i*_12_). As observed in Figure 3B for an 8oxoG DNA lesion, synthesis of the full-length (62-mer) primer extension product is clearly observed, indicating that human pol δ supports stable incorporation of dNTPs opposite and beyond an 8oxoG (i.e. lesion bypass) during an initial encounter. The observed *P*_*i*_ values up to, but not including, the 12^th^ dNTP incorporation step (*i*_12_) are identical for the native and 8oxoG P/T DNA substrates (Figure 3C). Thus, a downstream 8oxoG does not affect the progression of pol δ holoenzymes towards the lesion. Upon encountering an 8oxoG, only 36.4 ± 1.5% of the progressing pol δ holoenzymes bypass the lesion prior to dissociation (Supplementary Table S1, Probability of bypass × 100%), which is significantly less than that observed for bypass of native G (97.1 ± 0.1%) in the same sequence context. The reduced efficiency of 8oxoG bypass (37.5 ± 1.5%, Figure 3D) is primarily due to reduced extension efficiency (43.7 ± 1.5%) following moderately efficient insertion opposite 8oxoG (85.7 ± 0.6%). Immediately following lesion bypass of 8oxoG (*i*_12_ and *i*_13_), the observed *P*_i_ values (from *i*_14_ to *i*_33_) are restored to those observed for the native G template (Figure 3B). Thus, after bypass of an 8oxoG, the offending lesion does not affect the progression of pol δ holoenzymes that continue downstream. Altogether, this indicates that an 8oxoG lesion only promotes dissociation of pol δ during lesion bypass (i.e. insertion and extension).

Next, we further assessed the effects of an 8oxoG on the progression of pol δ holoenzymes that encounter the lesion. To do so, we calculated and directly compared the distributions of pol δ dissociation events that occur after an 8oxoG or a native G is encountered at *i*_12_ (Figure 3E). For pol δ holoenzymes that encounter a native G at *i*_12_ (‘G at *i*_12_’ in Figure 3E), the vast majority of the associated pol δ (74.7 ± 2.0%) does not dissociate at all before reaching the end of the template (*i*_32_ and *i*_33_), as expected. Furthermore, of the dissociation events that do occur, nearly all are observed during elongation. For pol δ holoenzymes that encounter an 8oxoG at *i*_12_ (‘8oxoG at *i*_12_’ in Figure 3E), only 15.8 ± 0.6% of pol δ dissociates during insertion, indicating that a very high proportion of 8oxoG lesions encountered by progressing pol δ holoenzymes (84.2 ± 0.6%) are replicated by pol δ. Dissociation of pol δ is most prevalent during extension (47.8 ± 0.8%) but a significant portion of pol δ (27.8 ± 1.6%) does not dissociate at all before reaching the end of the template.

**Figure 4.**
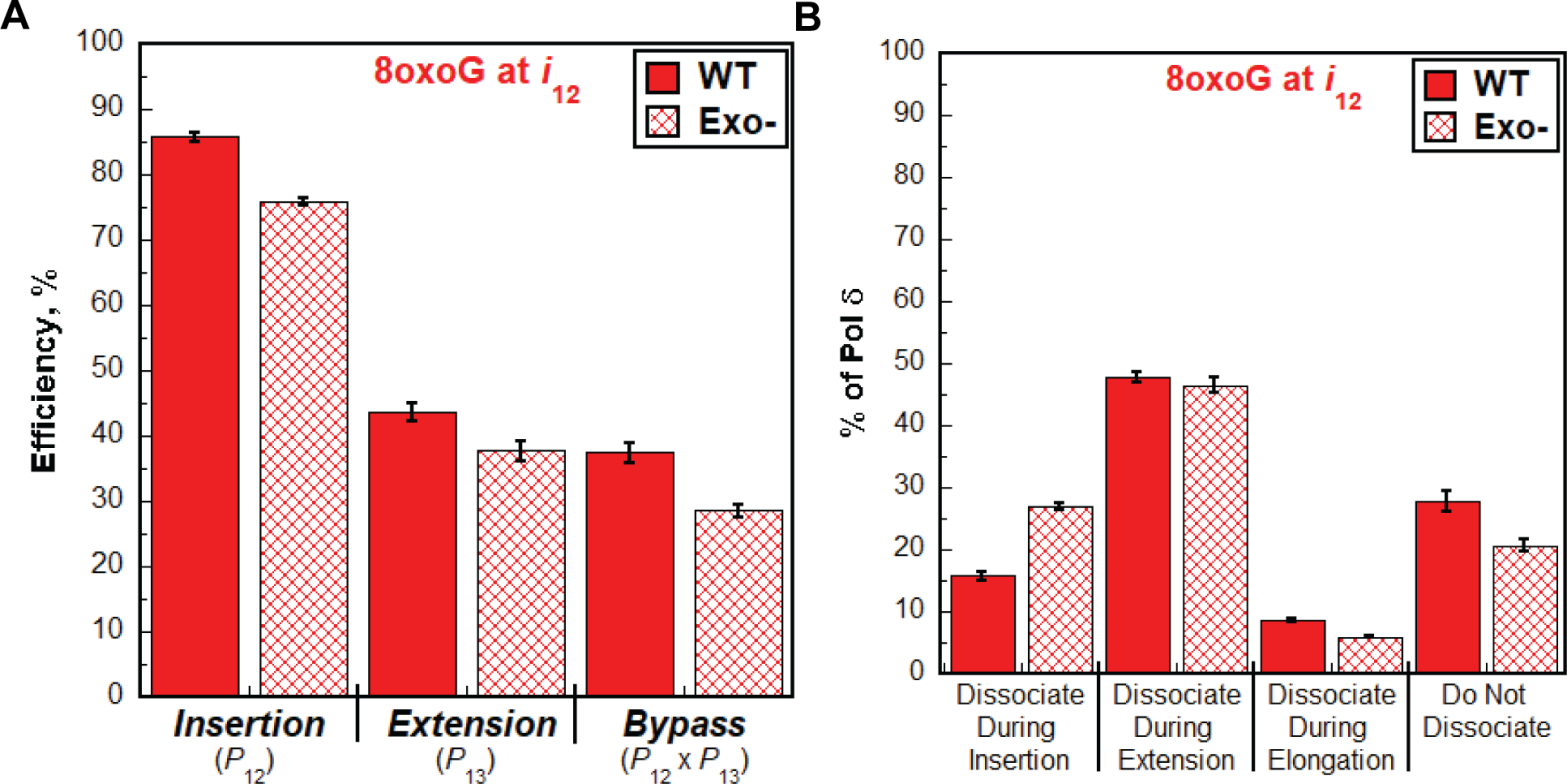
Effect of proofreading on bypass of 8oxoG by pol δ holoenzymes. (**A**) Efficiency of replicating 8oxoG. The efficiencies for insertion, extension, and bypass for wild-type (WT) and exonuclease-deficient (Exo–) pol δ holoenzymes are plotted as percentages. Values for each parameter are also reported in Supplementary Table S1. (**B**) Dissociation of pol δ holoenzymes after encountering an 8oxoG lesion at *i*_12_. The distribution of dissociation events observed for the Bio-Cy5-P/T-8oxoG DNA substrate with wild-type (WT) and exonuclease-deficient (Exo–) pol δ holoenzymes are plotted.

**Figure 5.**
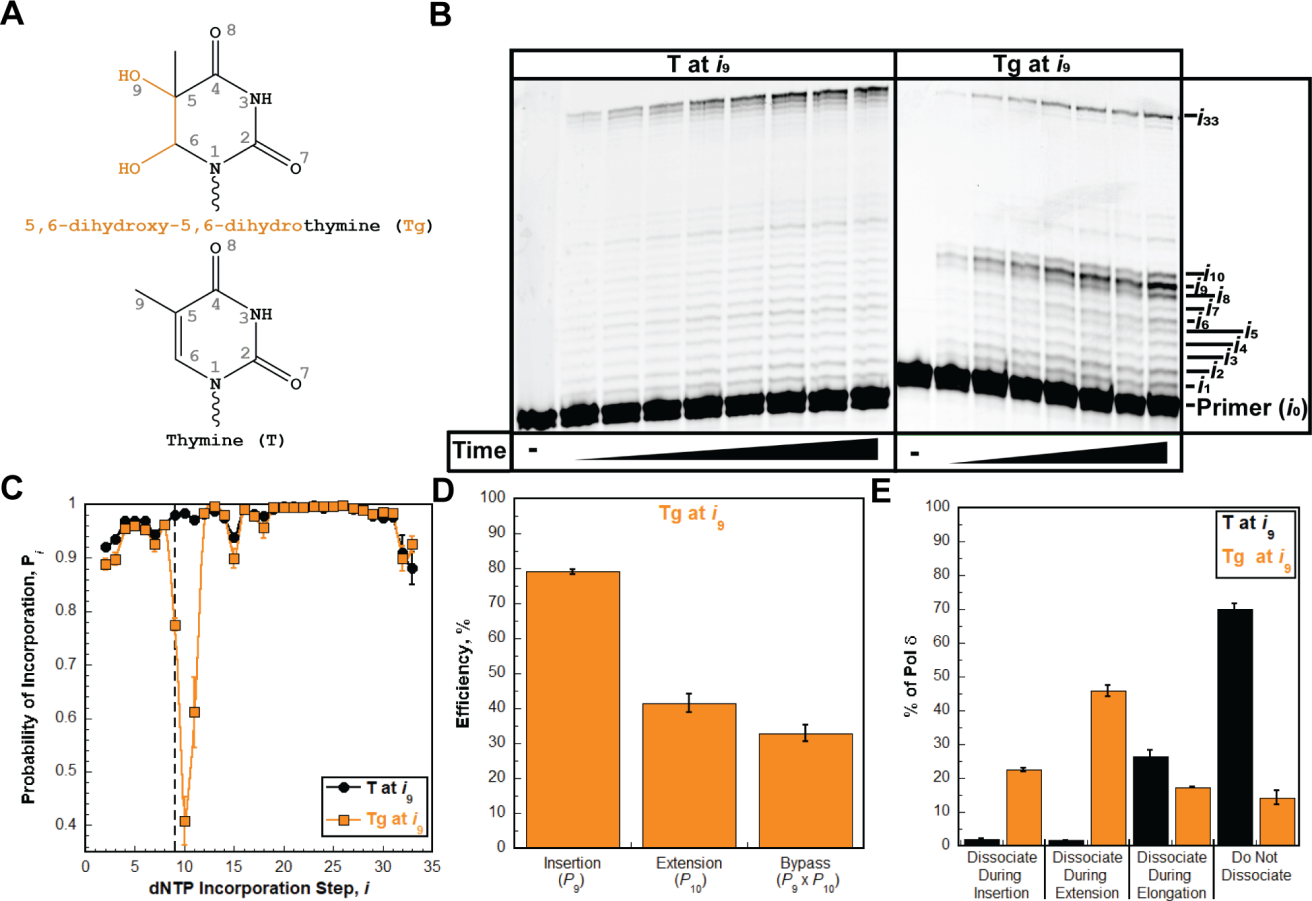
Pol δ holoenzymes encountering a Tg lesion downstream of a P/T junction. The progression of human pol δ holoenzymes was monitored on a P/T DNA substrate (Bio-Cy5-P/T-Tg, Supplementary Figure S1) that contains a Tg 9 nt downstream of the P/T junction (at the 9th dNTP incorporation step, *i*_9_). (**A**) Structure of Tg. Tg (bottom) is generated from T (top) through the addition of hydroxyl groups on the carbons at position 5 and position 6 of the ring. This results in a loss of aromaticity and conversion from planar to nonplanar. These modifications are highlighted in orange on the structure of Tg. (**B**) 16% denaturing sequencing gel of the primer extension products. Shown on the left and the right are representative gels of primer extension by pol δ holoenzymes on the native Bio-Cy5-P/T (‘T at *i*_9_’) and the Bio-Cy5-P/T-Tg (‘Tg at *i*_9_’) DNA substrates, respectively. (**C**) Processivity of pol δ holoenzymes. *P*_*i*_ values observed for the native Bio-Cy5-P/T (‘T at *i*_9_’) and the Bio-Cy5-P/T-Tg (‘Tg at *i*_9_’) DNA substrates are shown in black and orange, respectively, and plotted as a function of the dNTP incorporation step, *i*. Data is fit to an interpolation only for observation. Dashed line indicates dNTP incorporation step for insertion (*i*_9_). (**D**) Efficiency of replicating Tg. The efficiencies for insertion, extension, and bypass are calculated as described in *Materials and Methods* and plotted as percentages. Values for each parameter are also reported in Supplementary Table S2. (**E**). Dissociation of pol δ holoenzymes after encountering a Tg lesion. The distribution of dissociation events observed for the native Bio-Cy5-P/T (‘T at *i*_9_’) and the Bio-Cy5-P/T-Tg (‘Tg at *i*_9_’) DNA substrates are indicated by black and orange, respectively.

The intrinsic 3′→5′ exonuclease (i.e. proofreading) activity of human pol δ may affect 8oxoG bypass. To examine this possibility, we repeated the assays and analyses described above with exonuclease-deficient human pol δ. Under the conditions of the assay, where only initial binding encounters of pol δ are monitored, any observed proofreading occurs *intrinsically* (as opposed to *extrinsically*) because a given dNTP incorporation and the subsequent proofreading of that dNTP incorporation are not separated by a dissociation event. If lesion bypass is restricted by the proofreading activity of pol δ, then disabling this activity will increase efficiency of lesion bypass by promoting insertion, extension, or both activities, resulting in an increase in the percentage of pol δ that does not dissociate and a shift of the observed dissociation events towards elongation (39–41). Conversely, if lesion bypass is promoted by the proofreading activity of pol δ, then disabling this activity will decrease the efficiency of lesion bypass by prohibiting insertion, extension, or both activities, resulting in a decrease in the percentage of pol δ that does not dissociate and a shift in the observed dissociation events towards insertion and/or extension. As observed in Figure 4A and Supplementary Table S1, disabling the proofreading activity of human pol δ marginally decreases the bypass efficiency (by 8.91 ± 1.84%) by slightly decreasing the insertion efficiency (by 9.88 ± 0.91%) and the extension efficiency (by 6.01 ± 2.07%). This results in a decrease in the percentage of pol δ that does not dissociate and a shift in dissociation events to insertion (Figure 4B). Altogether, the studies described above suggest that human pol δ holoenzymes are very efficient at insertion opposite 8oxoG in a lagging strand template (insertion efficiency = 85.7 ± 0.6%) and that 8oxoG only promotes dissociation of pol δ during lesion bypass; 8oxoG does not affect the progression of pol δ holoenzymes towards the lesion (i.e. before lesion bypass) or 2 nt beyond the lesion (i.e. after lesion bypass). Furthermore, the 3′→5′ exonuclease activity of human pol δ marginally promotes 8oxoG bypass by proofreading insertion opposite the lesion and potentially extension beyond the lesion. Under the conditions of the assay, it cannot be discerned whether the contribution of proofreading to extension is due to proofreading insertion of an incorrect dNTP opposite 8oxoG (i.e. a mismatch) to promote extension or to proofreading extension to stabilize dNTP incorporation 1 nt downstream of the lesion. Next, we repeated these assays to analyze the effects of another prominent oxidative DNA lesion, 5,6-dihydroxy-5,6-dihydrothymine, i.e. thymine glycol (Tg, Figure 5A), on the progression of human pol δ holoenzymes (19). Thymine glycol is the most common oxidation product of thymine (19).

The P/T DNA substrate (Bio-Cy5-P/T-Tg, Supplementary Figure S1) contains a Tg 9 nt downstream of the P/T junction (at the 9^th^ dNTP incorporation step, *i*_9_). As observed in Figure 5B, synthesis of the full-length (62-mer) primer extension product is clearly observed, indicating that human pol δ supports lesion bypass of a Tg during an initial encounter. The observed *P*_*i*_ values up to, but not including, the 9th dNTP incorporation step (*i*_9_) are nearly identical for the native and Tg P/T DNA substrates (Figure 5C). Thus, a downstream Tg does not significantly affect, if at all, the progression of pol δ holoenzymes towards the lesion. Upon encountering a Tg, only 31.6 ± 2.3% of replicating pol δ holoenzymes bypass the lesion prior to dissociation, which is significantly less than that observed for bypass of native T (96.3 ± 0.2%) in the same sequence context (Supplementary Table S2). The reduced efficiency (32.9 ± 2.4%) of Tg bypass (Figure 5D) is primarily due to reduced extension efficiency (41.5 ± 2.7%) following moderately efficient insertion opposite Tg (79.1 ± 0.7%). Immediately following lesion bypass of Tg, native *P_i_* values are not restored until 3 nt beyond the lesion (at the 12th dNTP incorporation step, Figure 5C). Thus, after bypass of a Tg, the offending lesion promotes dissociation of pol δ holoenzymes that continue downstream. Altogether, this indicates that a Tg lesion promotes dissociation of pol δ during insertion, extension, and the first dNTP incorporation step of elongation (i.e. *i*_11_). However, only 22.5 ± 0.7% of pol δ dissociates during insertion (Figure 5E), indicating that a very high proportion of Tg lesions encountered by progressing pol δ holoenzymes (77.5 ± 0.7%) are replicated by this DNA polymerase. Dissociation of pol δ is most prevalent during extension (45.9 ± 1.7%) but a significant portion (17.4 ± 0.3%) dissociates during elongation, primarily at *i*_11_. Furthermore, 14.3 ± 2.1% of pol δ does not dissociate at all before reaching the end of the template.

**Figure 6.**
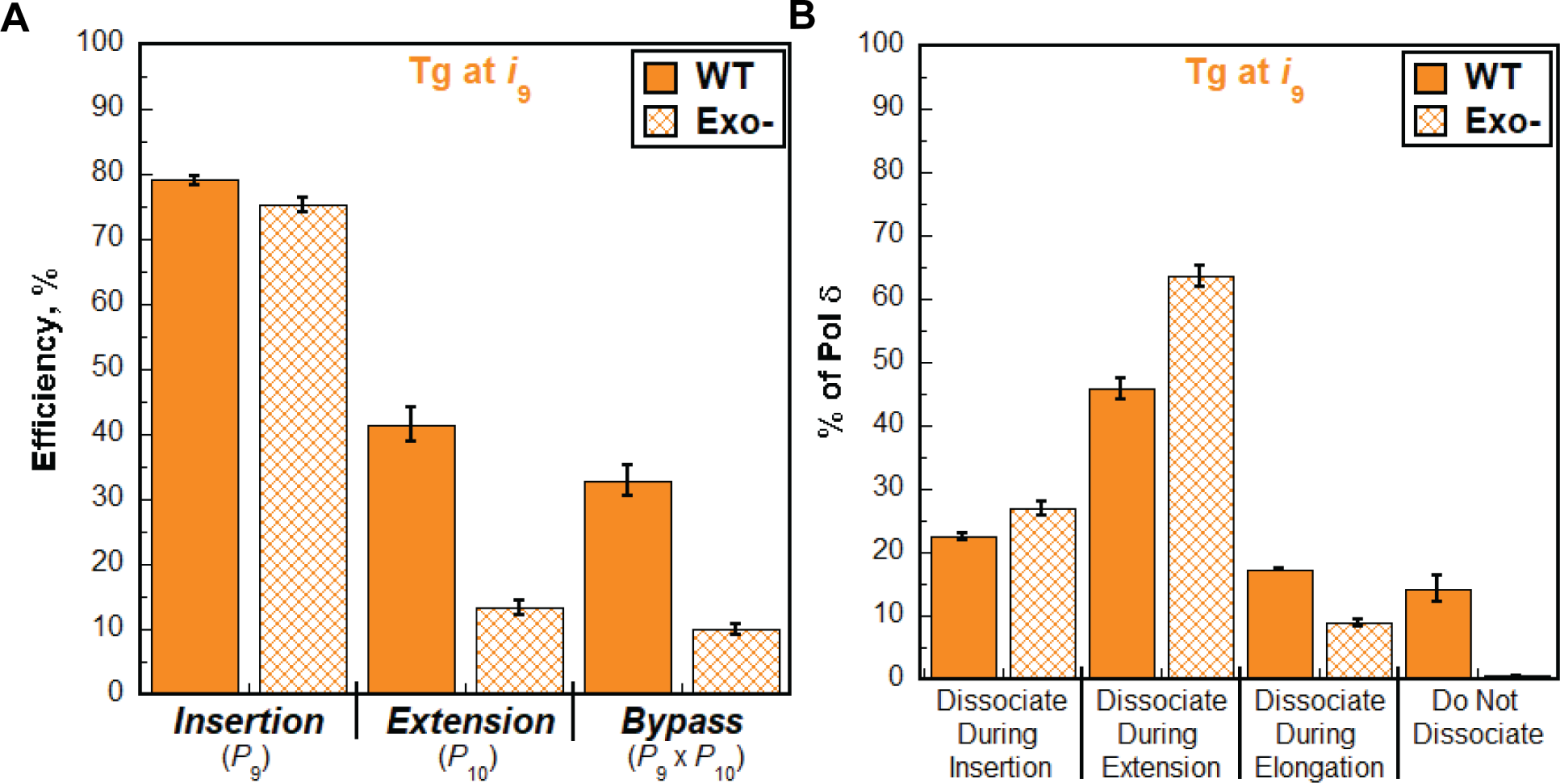
Effect of proofreading on bypass of Tg by pol δ holoenzymes. (**A**) Efficiency of replicating Tg. The efficiencies for insertion, extension, and bypass for wild-type (WT) and exonuclease-deficient (Exo–) pol δ holoenzymes are plotted as percentages. Values for each parameter are also reported in Supplementary Table S2. (**B**) Dissociation of pol δ holoenzymes after encountering an Tg lesion. The distribution of dissociation events observed for the Bio-Cy5-P/T-Tg DNA substrate with wild-type (WT) and exonuclease-deficient (Exo–) pol δ holoenzymes are plotted.

As observed in Figure 6A, disabling the 3′→5′ exonuclease activity of human pol δ significantly decreases the bypass efficiency (by 22.9 ± 2.5%) primarily by decreasing the extension efficiency (by 28.2 ± 2.9%); the insertion efficiency is only reduced by 3.73 ± 1.32%. This results in a significant decrease in the percentage of pol δ that does not dissociate and a shift in dissociation events primarily to extension (Figure 6B). Altogether, the studies described above suggest that human pol δ holoenzymes are very efficient at insertion opposite Tg in a lagging strand template (insertion efficiency = 79.1 ± 0.7%) and that Tg does not affect the progression of pol δ holoenzymes towards the lesion (i.e. before lesion bypass) but promotes dissociation of pol δ during and after lesion bypass. Furthermore, the 3′→5′ exonuclease activity of human pol δ significantly promotes Tg bypass primarily by promoting extension. Again, under the conditions of the assay, it cannot be discerned whether the significant contribution of proofreading to extension is due to proofreading insertion of an incorrect dNTP opposite a Tg (i.e. a mismatch) to promote extension or proofreading extension to stabilize dNTP incorporation 1 nt downstream of the lesion. Next, we examined the effects of prominent alkylative DNA lesions on the progression of human pol δ holoenzymes.

### The effect of alkylative DNA lesions on the progression of human pol δ holoenzymes

**Figure 7.**
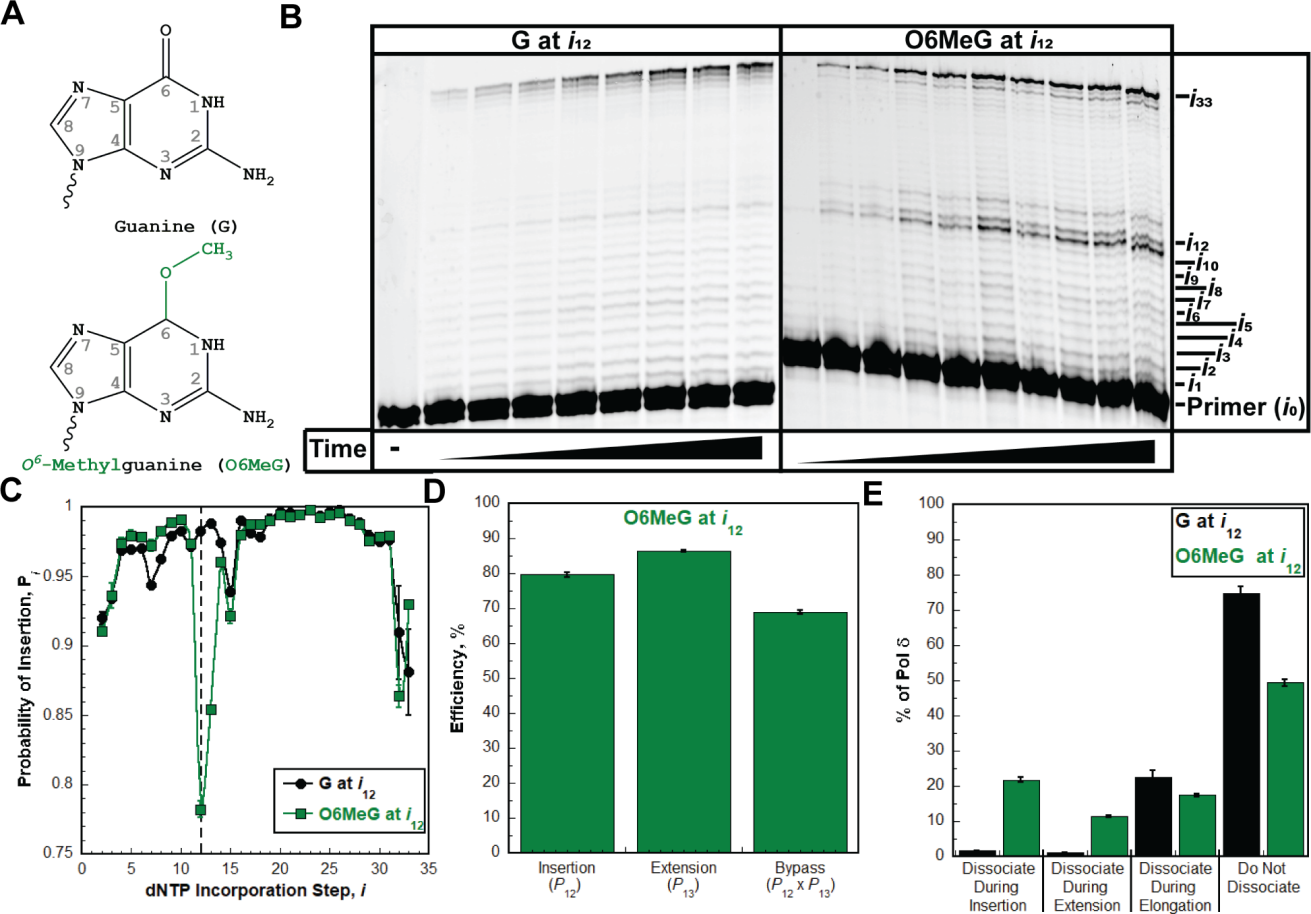
Pol δ holoenzymes encountering an O6MeG lesion downstream of a P/T junction. The progression of human pol δ holoenzymes was monitored on a P/T DNA substrate (Bio-Cy5-P/T-O6MeG, Supplementary Figure S1) that contains an O6MeG 12 nt downstream of the P/T junction (at the 12th dNTP incorporation step, *i*_12_). (**A**) Structure of O6MeG. O6MeG (bottom) is generated from G (top) through the addition of a methyl group on the oxygen of the carbonyl group at position 6 of the ring. These modifications are highlighted in green on the structure of O6MeG. (**B**) 16% denaturing sequencing gel of the primer extension products. Shown on the left and the right are representative gels of primer extension by pol δ holoenzymes on the native Bio-Cy5-P/T (‘G at *i*_12_’) and the Bio-Cy5-P/T-O6MeG (‘O6MeG at *i*_12_’) DNA substrates, respectively. (**C**) Processivity of pol δ holoenzymes. *P*_i_ values observed for the native Bio-Cy5-P/T (‘G at *i*_12_’) and the Bio-Cy5-P/T-O6MeG (‘O6MeG at *i*_12_’) DNA substrates are shown in black and green, respectively, and plotted as a function of the dNTP incorporation step, *i*. Data is fit to an interpolation only for observation. Dashed line indicates dNTP incorporation step for insertion (*i*_12_). (**D**) Efficiency of replicating O6MeG. The efficiencies for insertion, extension, and bypass are calculated as described in *Materials and Methods* and plotted as percentages. Values for each parameter are also reported in Supplementary Table S3. (**E**). Dissociation of pol δ holoenzymes after encountering an O6MeG lesion. The distribution of dissociation events observed for the native Bio-Cy5-P/T (‘G at *i*_12_’) and the Bio-Cy5-P/T-O6MeG (‘O6MeG at *i*_12_’) DNA substrates are indicated by black and green, respectively.

First, we examined the effect of *O^6^*-Methylguanine (O6MeG, Figure 7A) on the progression of human pol δ holoenzymes. O6MeG is a prominent DNA lesion generated by exposure of genomic DNA to methylating agents, such as the antitumor agents dacarbazine, streptozotocin, procarbazine and temozolomide (42). The P/T DNA substrate (Bio-Cy5-P/T-O6MeG, Supplementary Figure S1) contains an O6MeG 12 nt downstream of the P/T junction (at the 12^th^ dNTP incorporation step, *i*_12_). As observed in Figure 7B, synthesis of the full-length (62-mer) primer extension product is clearly observed, indicating that human pol δ supports stable incorporation of dNTPs opposite and beyond an O6MeG (i.e. lesion bypass) during an initial encounter. The observed *P*_i_ values up to, but not including, the 12^th^ dNTP incorporation step (*i*_12_) are essentially identical for the native and O6MeG P/T DNA substrates (Figure 7C). Thus, a downstream O6MeG DNA lesion does not affect the progression of pol δ holoenzymes towards the lesion. Upon encountering an O6MeG, 66.8 ± 0.6% of replicating pol δ holoenzymes bypass the lesion prior to dissociation, which is reduced compared to that observed for bypass of native G (97.1 ± 0.1%) in the same sequence context (Supplementary Table S3). The marginally reduced efficiency of O6MeG bypass (68.8 ± 0.6%, Figure 7D, Supplementary Table S3) is due to moderate reductions in both the insertion (79.6 ± 0.6%) and extension efficiencies (86.5 ± 0.3%). Immediately following O6MeG bypass, native *P*_i_ values are restored (at the 14th dNTP incorporation step, *i*_14_, Figure 7C), indicating that, after bypass of an O6MeG, the offending lesion does not affect the progression of pol δ holoenzymes that continue downstream. Altogether, this indicates that an O6MeG lesion only promotes dissociation of pol δ during lesion bypass (i.e. insertion and extension). However, only 21.8 ± 0.6% of pol δ dissociates during insertion (Figure 7E), indicating that a very high proportion of O6MeG lesions encountered by progressing pol δ holoenzymes (78.2 ± 0.6%) are replicated by pol δ. Dissociation of pol δ is less prevalent during extension (11.4 ± 0.2%) and elongation (17.5 ± 0.4%) and, surprisingly, half of pol δ (49.3 ± 0.9%) does not dissociate at all before reaching the end of the template.

**Figure 8.**
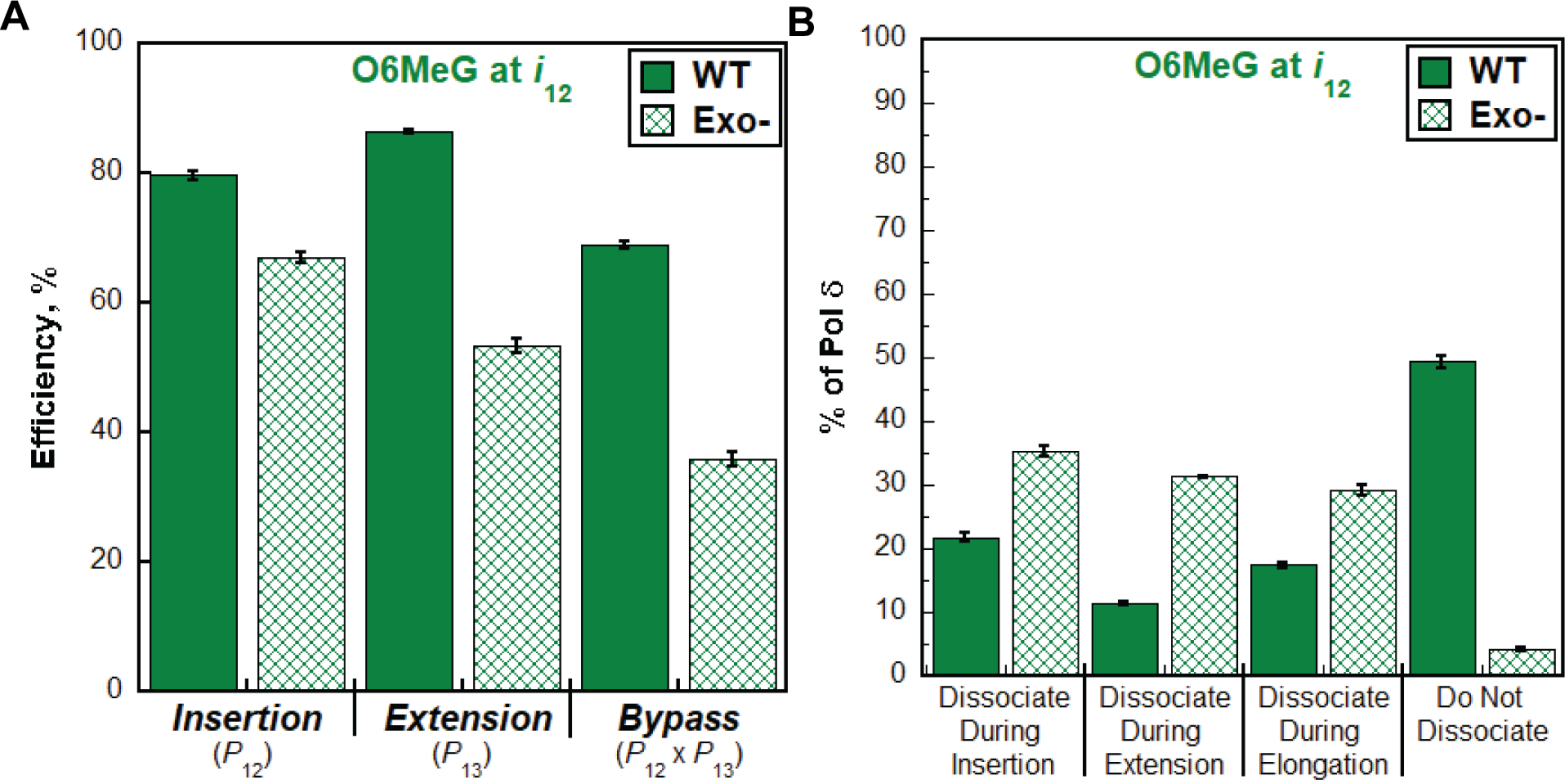
Effect of proofreading on bypass of O6MeG by pol δ holoenzymes. (**A**) Efficiency of replicating O6MeG. The efficiencies for insertion, extension and bypass for wild-type (WT) and exonuclease-deficient (Exo–) pol δ holoenzymes are plotted as percentages. Values for each parameter are also reported in Supplementary Table S3. (**B**) Dissociation of pol δ holoenzymes after encountering an O6MeG lesion. The distribution of dissociation events observed for the Bio-Cy5-P/T-O6MeG DNA substrate with wild-type (WT) and exonuclease-deficient (Exo–) pol δ holoenzymes are plotted.

**Figure 9.**
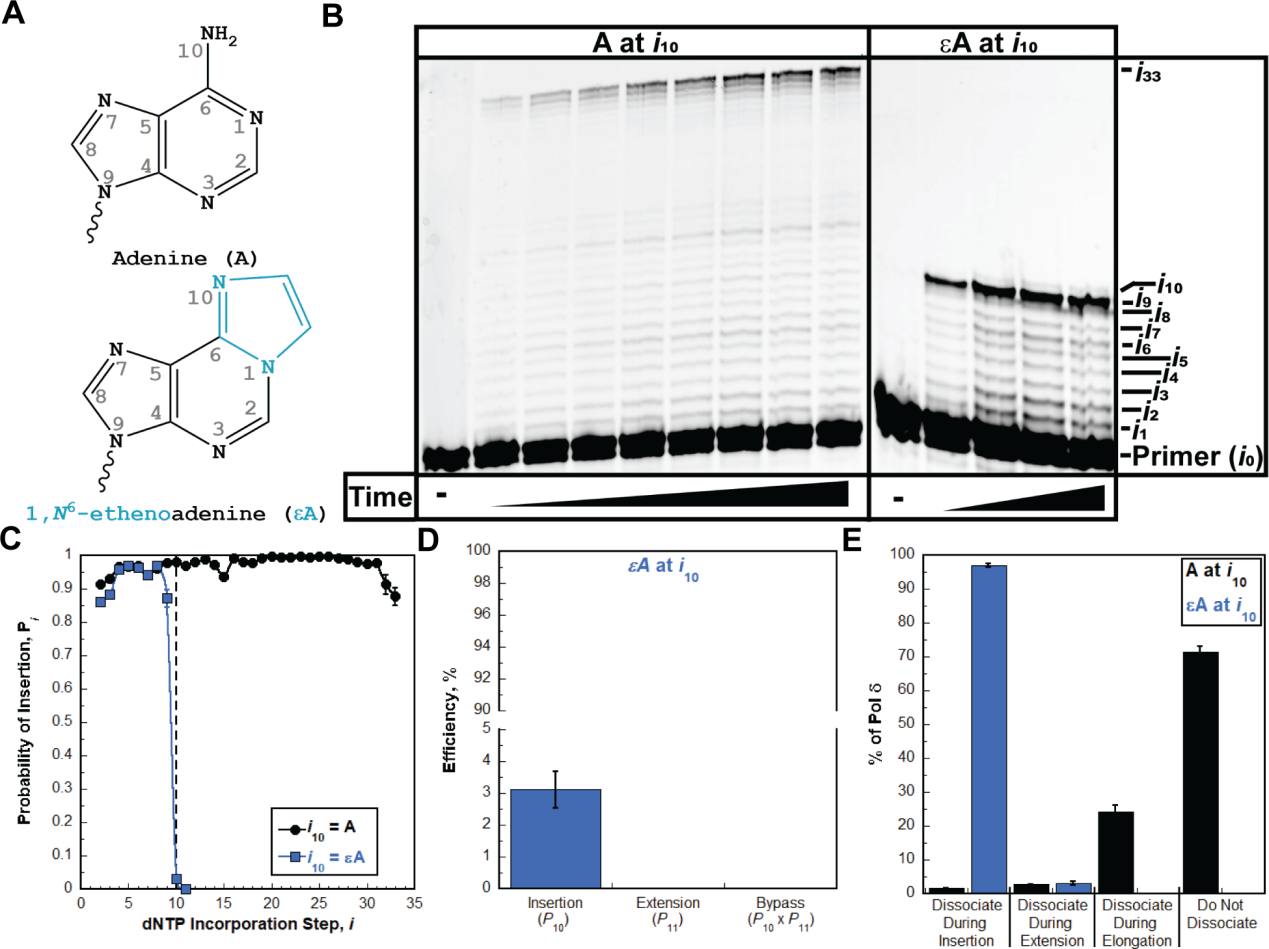
Pol δ holoenzymes encountering an ϵA lesion downstream of a P/T junction. The progression of human pol δ holoenzymes was monitored on a P/T DNA substrate (Bio-Cy5-P/T-ϵA, Supplementary Figure S1) that contains an ϵA 10 nt downstream of the P/T junction (at the 10th dNTP incorporation step, *i*_10_). (**A**) Structure of ϵA. ϵA (bottom) is generated from A (top) through the attachment of two extra carbons in an exocyclic arrangement; 1 carbon is attached to the nitrogen at position 1 and the other is attached to the nitrogen in the amine at position 6 of the ring. These modifications are highlighted in blue on the structure of ϵA. (**B**) 16% denaturing sequencing gel of the primer extension products. Shown on the left and the right are representative gels of primer extension by pol δ holoenzymes on the native Bio-Cy5-P/T (‘A at *i*_10_’) and the Bio-Cy5-P/T-ϵA (‘ϵA at *i*_10_’) DNA substrates, respectively. (**C**) Processivity of pol δ holoenzymes. *P*_i_ values observed for the native Bio-Cy5-P/T (‘A at *i*_10_’) and the Bio-Cy5-P/T-ϵA (‘ϵA at *i*_10_’) DNA substrates are shown in black and blue, respectively, and plotted as a function of the dNTP incorporation step, *i*. Data is fit to an interpolation only for observation. (**D**) Efficiency of replicating ϵA. The efficiencies for insertion, extension and bypass are calculated as described in *Materials and Methods* and plotted as percentages. Values for each parameter are also reported in Supplementary Table S4. (**E**). Dissociation of pol δ holoenzymes after encountering an ϵA lesion. The distribution of dissociation events observed for the native Bio-Cy5-P/T (‘A at *i*_10_’) and the Bio-Cy5-P/T-ϵA (‘ϵA at *i*_10_’) DNA substrates are indicated by black and blue, respectively.

As observed in Figure 8A, disabling the 3′→5′ exonuclease activity of human pol δ significantly decreases the bypass efficiency for O6MeG (by 33.0 ± 1.3%) by slightly decreasing the insertion efficiency (by 12.6 ± 1.0%) and significantly decreasing the extension efficiency (by 33.1 ± 1.0%). This results in a drastic decrease in the percentage of pol δ that does not dissociate (Figure 8B) and a significant increase in the percentage of pol δ that dissociates during insertion (21.8 ± 0.6% to 35.4 ± 0.8%), extension (11.4 ± 0.2% to 31.3 ± 0.2%), as well as elongation (17.5 ± 0.4% to 29.2 ± 0.8%). Altogether, these studies suggest that human pol δ holoenzymes are very efficient at insertion opposite O6MeG in a lagging strand template (insertion efficiency = 79.6 ± 0.6%) and that O6MeG only promotes dissociation of pol δ during lesion bypass; O6MeG does not affect the progression of pol δ holoenzymes towards the lesion (i.e. before lesion bypass) or 2 nt beyond the lesion (i.e. after lesion bypass). Furthermore, the 3′→5′ exonuclease activity of human pol δ significantly promotes O6MeG bypass by proofreading insertion opposite the lesion and potentially extension beyond the lesion. Again, under the conditions of the assay, it cannot be discerned whether the significant contribution of proofreading to extension is due to proofreading insertion of an incorrect dNTP opposite O6MeG (i.e. a mismatch) to promote extension or proofreading extension to stabilize dNTP incorporation 1 nt downstream of the lesion. Finally, we repeated these assays to analyze the effects of another prominent alkylative DNA lesion, *1,N^6^*-ethenoadenine (ϵA, Figure 9A) on the progression of human pol δ holoenzymes. ϵA is a prominent alkylation product of adenine that is generated by exposure of genomic DNA to vinyl chloride, an industrial pollutant, or lipid peroxidation byproducts associated with inflammation and metabolism (43).

**Figure 10.**
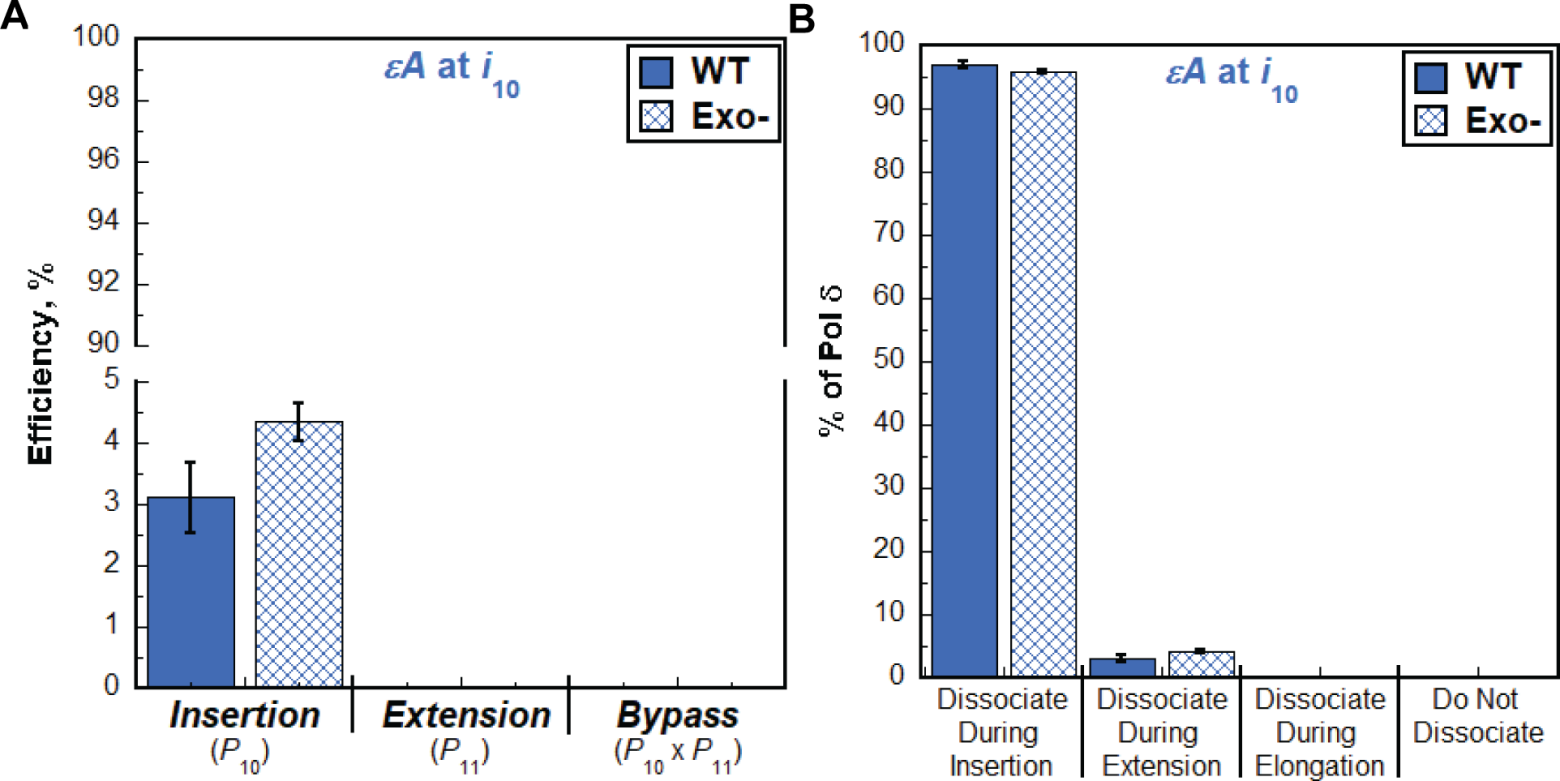
Effect of proofreading on bypass of ϵA by pol δ holoenzymes. (**A**) Efficiency of replicating ϵA. The efficiencies for insertion, extension, and bypass for wild-type (WT) and exonuclease-deficient (Exo-) pol δ holoenzymes are plotted as percentages. Values for each parameter are also reported in Supplementary Table S4. (**B**) Dissociation of pol δ holoenzymes after encountering an ϵA lesion. The distribution of dissociation events observed for the Bio-Cy5-P/T-ϵA DNA substrate with wild-type (WT) and exonuclease-deficient (Exo-) pol δ holoenzymes are plotted.

The P/T DNA substrate (Bio-Cy5-P/T-ϵA, Supplementary Figure S1) contains an ϵA 10 nt downstream of the P/T junction (at the 10th dNTP incorporation step, *i*_10_). As observed in Figure 9B, synthesis past the ϵA DNA lesion does not occur, indicating that human pol δ does not support lesion bypass of an ϵA during an initial encounter. Interestingly, the observed *P*_i_ values for the native and ϵA P/T DNA substrates are nearly identical up to only the 8^th^ dNTP incorporation step (*i*_8_) and then significantly diverge at the 9^th^ dNTP incorporation step (*i*_9_) and beyond (Figure 9C). This suggests that a downstream ϵA may cause some progressing pol δ holoenzymes to prematurely dissociate before the lesion is encountered. Upon encountering an ϵA lesion, only 3.06 ± 0.56% of progressing pol δ holoenzymes incorporate a dNTP opposite the lesion prior to dissociation, which is drastically less than that observed for bypass of native A (98.3 ± 0.1%) in the same sequence context (Supplementary Table S4). Hence, the efficiency for dNTP incorporation opposite an ϵA lesion is extremely low (insertion efficiency = 3.11 ± 0.57%, Figure 9D) and by far the lowest of all DNA lesions analyzed in the present study. Extension beyond a ϵA lesion and, hence, bypass and elongation are not observed. Thus, all progressing pol δ holoenzymes that encounter an ϵA lesion dissociate during insertion or extension (Figure 9E). The former accounts for 96.9 ± 0.6% of all dissociation events. As observed in Figure 10A, disabling the 3′→5′ exonuclease activity of human pol δ slightly increased the insertion efficiency, if at all, and did not yield extension. Furthermore, the observed distribution of dissociation events is not visibly altered by disabling the 3′→5′ exonuclease activity of human pol δ (Figure 10B). This suggests that the 3′→5′ exonuclease activity of human pol δ does not contribute to dNTP incorporation opposite ϵA and that insertion may evade intrinsic proofreading by human pol δ. Altogether, these studies suggest that human pol δ holoenzymes are very inefficient at insertion opposite an ϵA in a lagging strand template (insertion efficiency = 3.11 ± 0.57%) and that ϵA promotes dissociation of pol δ during lesion bypass and also as the polymerase approaches the lesion. Furthermore, the 3′→5′ exonuclease activity of human pol δ does not contribute to lesion bypass of ϵA during initial encounters.

## DISCUSSION

**Figure 11.**
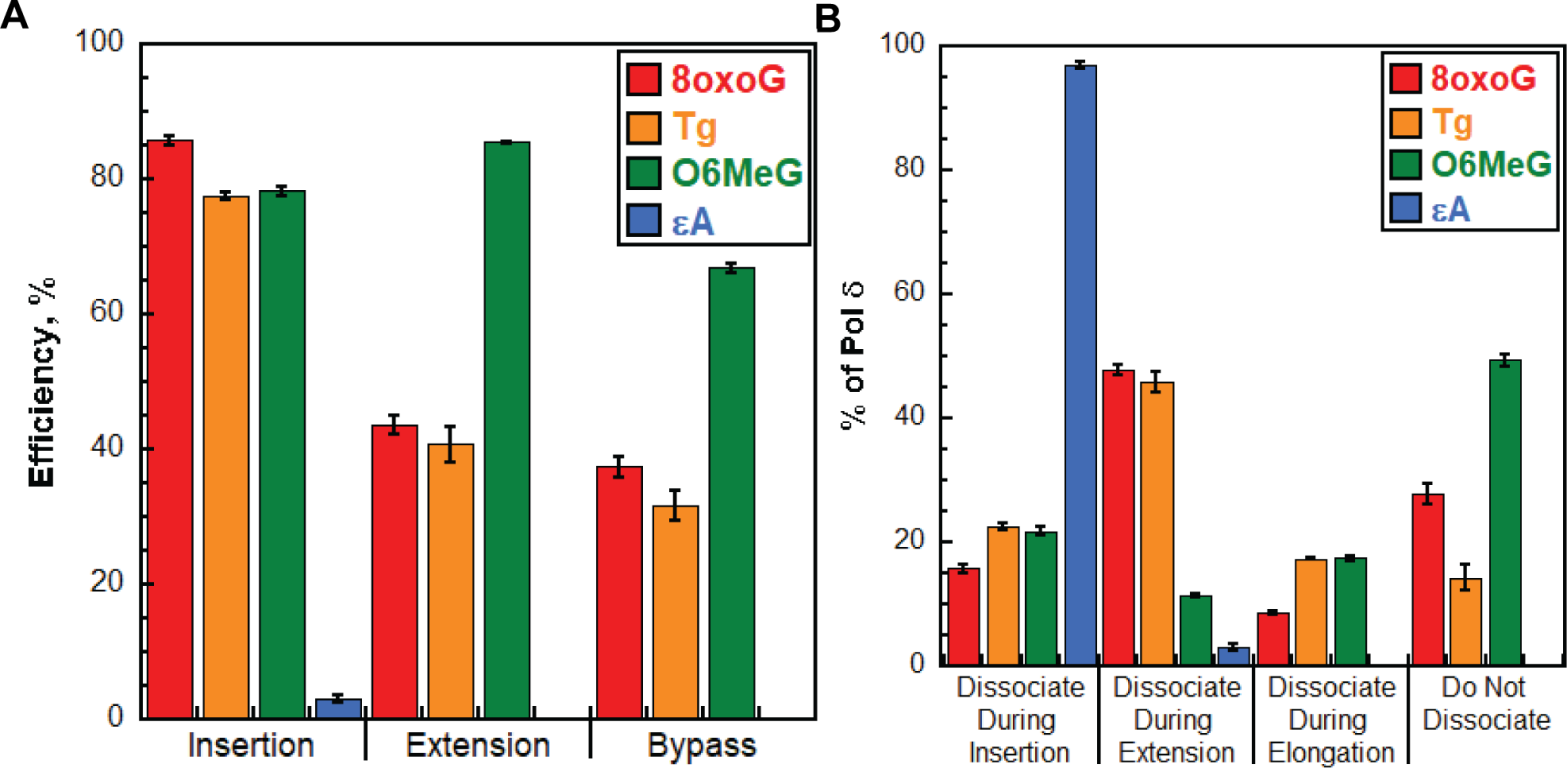
Bypass of DNA lesions by pol δ holoenzymes during initial encounters. (**A**) Efficiencies of replicating DNA lesions. The efficiencies for dNTP incorporation opposite a lesion (i.e. insertion), 1 nt downstream of lesion (i.e. extension) and bypass (insertion and extension) of a lesion for wild type pol δ holoenzymes are plotted. Data is taken from Figures 3D, 5D, 7D and 9D and is color-coded by DNA lesion. (**B**) Dissociation of pol δ holoenzymes after encountering DNA lesions. The distribution of dissociation events observed for wild type pol δ holoenzymes encountering DNA lesions is plotted. Data is taken from Figures 3E, 5E, 7E and 9E and is color-coded by DNA lesion.

In the present study, we re-constituted human lagging strand replication at physiological pH, ionic strength, and dNTP concentrations to quantitatively characterize, at single nucleotide resolution, the initial encounters of pol δ holoenzymes with downstream DNA lesions. In short, a DNA lesion ≥9 nt downstream of a P/T junction is encountered only once and only by a progressing pol δ holoenzyme, rather than pol δ alone. To the best of our knowledge, comparable studies of human lagging strand replication have yet to be reported. The results indicate that human pol δ holoenzymes support stable dNTP incorporation opposite and beyond multiple lesions and the extent of these activities depends on the identity of the lesion (Figure 11A) and the ability to proofread intrinsically. Surprisingly, the results reveal that human pol δ holoenzymes are very efficient at inserting a dNTP opposite certain DNA lesions, with insertion efficiencies ≥∼80% for 8oxoG, Tg and O6MeG lesions (Figure 11A). Furthermore, the results indicate that after a progressing pol δ holoenzyme encounters a given DNA lesion, subsequent dissociation of pol δ, if it occurs, does not converge to a uninform site relative to the lesion. Rather, pol δ dissociation events are distributed around the lesion. The distributions of pol δ dissociation events are dependent on the identity of the lesion (Figure 11B) and the ability of pol δ to proofread intrinsically. Taken together with previous reports on human pol δ, the results from the present study reveal complexity and heterogeneity in the replication of DNA lesions in lagging strand templates, as discussed in further detail below.

### 8oxoG lesions in lagging strand templates

Human pol δ holoenzymes are very efficient at inserting a dNTP opposite 8oxoG (insertion efficiency = 85.7 ± 0.6%, Figure 11A) such that 84.2 ± 0.6% of progressing pol δ holoenzymes that encounter an 8oxoG lesion complete insertion prior to pol δ dissociating (Figure 11B). Intrinsic proofreading contributes marginally (9.88 ± 0.91%) to this high insertion efficiency (Figure 4A). This suggests that a very high proportion of 8oxoG lesions in lagging strand templates are *initially* replicated by pol δ, rather than a DDT pathway such as TLS. Previous studies indicated that, in the presence of PCNA and RPA, human pol δ primarily inserts either the correct dCTP or the incorrect dATP opposite 8oxoG, with the latter accounting for 25–40% of all insertion events (9,35,44). In the present study, this equates to ∼21–34% of all encounters between progressing pol δ holoenzymes and 8oxoG lesions resulting in 8oxoG:A mismatches and ∼50–63% yielding ‘correct’ 8oxoG:C base pairs.

Interestingly, human pol δ holoenzymes are ∼2-fold more efficient at insertion opposite 8oxoG (insertion efficiency = 85.7 ± 0.6%, Figure 11A) than extension of 8oxoG base pairs (extension efficiency = 43.7 ± 1.5%, Figure 11A) such that only 43.2 ± 1.4% of progressing pol δ holoenzymes that complete insertion opposite 8oxoG subsequently complete extension prior to pol δ dissociating (Supplementary Table S1). Intrinsic proofreading contributes marginally (6.01 ± 2.07%) to the observed extension efficiency (Figure 4A). Consequently, only 36.4 ± 1.5% of progressing pol δ holoenzymes that encounter an 8oxoG complete lesion bypass prior to pol δ dissociating (Supplementary Table S1). Previous qualitative studies demonstrated that, in the presence of PCNA and RPA, human pol δ has a slight but visible preference for extending 8oxoG:A mismatches compared to 8oxoG:C (11). The extent of this preference has yet to be quantified. Nonetheless, the (in)fidelity of insertion opposite 8oxoG (discussed above) together with the preferential extension of 8oxoG base pairs may explain the reduced efficiencies for extension and lesion bypass observed in the present study; human pol δ holoenzymes preferentially insert dCTP opposite 8oxoG but are inefficient at extending 8oxoG:C base pairs.

Collectively, the results presented here suggest that a very high proportion of 8oxoG lesions in lagging strand templates (84.2 ± 0.6%) are *initially* replicated by pol δ, rather than a DDT pathway, creating a heterogenous population of nascent DNA that may elicit a variety of downstream responses during DNA replication. For 8oxoG:C base pairs, pol δ faithfully completes insertion and DDT would only be utilized, if at all, to complete extension as dissociation of pol δ is most prevalent at this dNTP incorporation step (Figure 11B) and unperturbed progression of pol δ holoenzymes resumes 2 nt downstream of the lesion (Figure 3C). For 8oxoG:A mismatches, the mismatched dAMP opposite the 8oxoG lesion must ultimately be excised and the 8oxoG accurately ‘re-replicated’ to avoid fixed G:C→T:A transversion mutations. This may occur via multiple pathways that depend on the extent of pol δ holoenzyme progression downstream of an 8oxoG lesion and/or the activation of DDT pathways. This is currently under investigation.

### Tg lesions in lagging strand templates

Similar to 8oxoG, human pol δ holoenzymes are very efficient at inserting a dNTP opposite Tg (insertion efficiency = 79.1 ± 0.7%, Figure 11A) such that 77.5 ± 0.7% of progressing pol δ holoenzymes that encounter a Tg lesion complete insertion prior to pol δ dissociating (Figure 11B). Furthermore, similar to 8oxoG, intrinsic proofreading slightly contributes (3.73 ± 1.32%) to this high insertion efficiency (Figure 6A). This suggests that a very high proportion of Tg lesions in lagging strand templates are *initially* replicated by pol δ, rather than a DDT pathway such as TLS. To the best of our knowledge, direct studies on replication of Tg lesions by human pol δ have yet to be reported and, hence, the fidelity of human pol δ in replicating Tg lesions is unknown. However, human DNA polymerase α (pol α), a B-family DNA polymerase like pol δ, exclusively inserts dAMP opposite Tg lesions (45). Furthermore, the DNA polymerase active sites of pol δ’s from *S. cerevisiae* and human are structurally conserved and a recent report demonstrated that *S. cerevisiae* pol δ exclusively inserts dAMP opposite Tg lesions in the presence of PCNA and RPA (46–48). Finally, previous structural studies of bacteriophage RB69 DNA polymerase, a B-family polymerase like pol δ, revealed that Tg is able to establish Watson-Crick base pair hydrogen bonds with an inserted dAMP, resembling a canonical T:A base pair. Altogether, this suggests that insertion opposite Tg lesions by human pol δ is error-free and the resultant Tg:A base pair resembles a canonical T:A base pair (49).

Similar to that observed for 8oxoG, human pol δ holoenzymes are ∼2-fold more efficient at insertion opposite Tg (insertion efficiency = 79.1 ± 0.7%, Figure 11A) than extending Tg base pairs (extension efficiency = 41.5 + 2.7%, Figure 11A) such that only 40.8 + 2.7% of progressing pol δ holoenzymes that complete insertion opposite Tg subsequently complete extension prior to pol δ dissociating (Supplementary Table S2). Intrinsic proofreading contributes significantly (28.2 ± 2.9%) to the observed extension efficiency (Figure 6A). Consequently, only 31.6% + 2.3% of progressing pol δ holoenzymes that encounter a Tg lesion complete lesion bypass prior to pol δ dissociating. The aforementioned structural studies of bacteriophage RB69 revealed that the nature of the template nucleobase immediately 5′ to the Tg lesion significantly influences extension and, hence, lesion bypass. Specifically, the C5 methyl group of Tg within a Tg:A base pair protrudes axially from its nonplanar pyrimidine ring and pushes the 5′ template nucleotide into an extrahelical position. These structural aberrations decrease the extension efficiency and are more pronounced for a larger 5′ purine (A or G) compared to a smaller 5′ pyrimidine (C or T) (49). In the present study, adenine is immediately 5′ to the Tg lesion and, hence, likely responsible for the significantly reduced efficiencies of extension and bypass observed in the present study.

Collectively, the results presented here suggest that a very high proportion of Tg lesions in lagging strand templates (77.5 ± 0.7%) are *initially* replicated by pol δ, rather than a DDT pathway, and that replication of Tg lesions by pol δ is error-free. Accordingly, DDT is primarily utilized, if at all, to complete extension and/or the 1^st^ dNTP incorporation of elongation as dissociation of pol δ is most prevalent during these dNTP incorporation steps and unperturbed progression of pol δ holoenzymes resumes 3 nt downstream of the lesion (Figure 5C). Utilization of DDT for extension may be promoted in the absence of intrinsic proofreading by pol δ as inactivation of the 3′→5′ exonuclease activity of human pol δ significantly decreases the extension efficiency (by 28.2 ± 2.9%, Figure 6A) leading to a significant increase (17.8 ± 2.4%, Figure 6B) in pol δ dissociation during this dNTP in corporation step.

### O6MeG in lagging strand templates

Similar to that observed for 8oxoG and Tg, human pol δ holoenzymes are also very efficient at inserting a dNTP opposite O6MeG (insertion efficiency = 79.6 ± 0.6%, Figure 11A) such that 78.2 ± 0.6% of progressing pol δ holoenzymes that encounter an O6MeG complete insertion prior to pol δ dissociating (Figure 11B). Furthermore, like 8oxoG and Tg, intrinsic proofreading visibly contributes (12.6 ± 1.0%) to this high insertion efficiency (Figure 8A). This suggests that a very high proportion of O6MeG lesions in lagging strand templates are *initially* replicated by pol δ, rather than a DDT pathway such as TLS. Previous studies indicated that, in the presence of PCNA and RPA, human pol δ primarily inserts either the correct dCTP or the incorrect dTTP opposite 8oxoG, and these events occur with equal probability (15). In the present study, this equates to ∼39.2% of all encounters between progressing pol δ holoenzymes and O6MeG lesions resulting in O6MeG:T mismatches and ∼39.2% yielding ‘correct’ O6MeG:C base pairs.

In contrast to that observed for 8oxoG and Tg, human pol δ holoenzymes are slightly more efficient at extension of O6MeG base pairs (extension efficiency = 86.5 ± 2.5%, Figure 11A) than insertion opposite O6MeG (insertion efficiency = 79.6 ± 0.6%, Figure 11A) such that 85.4 ± 0.2% of progressing pol δ holoenzymes that complete insertion opposite O6MeG subsequently complete extension prior to pol δ dissociating (Supplementary Table S3). Intrinsic proofreading contributes significantly (33.1 ± 1.0%) to the observed extension efficiency (Figure 8A). Consequently, 66.8% + 0.6% of progressing pol δ holoenzymes that encounter an O6MeG lesion complete lesion bypass prior to pol δ dissociating. Furthermore, over 60% (63.1 + 0.8%) of progressing pol δ holoenzymes that complete insertion opposite O6MeG subsequently extend the nascent DNA at least 19 nt downstream prior to pol δ dissociation. Together, this suggests that the majority of O6MeG in lagging strand templates and the corresponding template DNA sequences 5′ to the offending lesion are replicated by human pol δ without activation of DDT.

Collectively, the results presented here suggest that a very high proportion of O6MeG lesions in lagging strand templates (78.2 ± 0.6%) are *initially* replicated by pol δ, rather than a DDT pathway, creating a heterogenous population of nascent DNA that may elicit a variety of downstream responses during DNA replication. For O6MeG:C base pairs, pol δ faithfully completes insertion and DDT would only be utilized, if at all, to complete extension as dissociation of pol δ is most prevalent at this dNTP incorporation step (Figure 11B) and unperturbed progression of pol δ holoenzymes resumes 2 nt downstream of the lesion (Figure 7C). Utilization of DDT for extension may be promoted in the absence of intrinsic proofreading by pol δ as inactivation of the 3′→5′ exonuclease activity of human pol δ significantly decreases the extension efficiency (by 33.1 ± 1.0%, Figure 8A) leading to a significant increase (19.9 ± 0.3%, Figure 8B) in pol δ dissociation during this dNTP incorporation steps. For O6MeG:T mismatches, the mismatched dTMP opposite the O6MeG lesion must ultimately be excised and the O6MeG accurately ‘re-replicated’ to avoid fixed G:C→A:T transversion mutations. This may occur via multiple pathways that depend on the extent of pol δ holoenzyme progression downstream of an O6MeG lesion and/or the activation of DDT pathways. This is currently under investigation.

### ϵA in lagging strand templates

In contrast to 8oxoG, Tg, and O6MeG, human pol δ holoenzymes are very inefficient at inserting a dNTP opposite ϵA (insertion efficiency = 3.11 ± 0.57, Figure 11A) such that only 3.06 ± 0.56% of progressing pol δ holoenzymes that encounter an ϵA complete insertion prior to pol δ dissociating (Figure 11B). Furthermore, human pol δ holoenzymes are incapable of extending from a dNMP inserted opposite ϵA. Finally, intrinsic proofreading by pol δ does not contribute to the aforementioned behaviors (Figure 10). Altogether, this suggests that ϵA are very strong blocks to pol δ holoenzyme progression and, consequently, nearly all ϵA lesions in lagging strand templates are replicated by a DDT pathway such as TLS. This agrees with a previous *ex vivo* study (50). It is also possible that upon dissociation of pol δ during insertion opposite ϵA or extension from an ϵA base pair, the offending lesion is subsequently repaired via direct reversal and the restored native adenine is then replicated by a pol δ holoenzyme (51–53). This is currently under investigation.

## DATA AVAILABILITY

Exact sequences, exact details of chemical modifications at any position, and source of synthetic nucleic acid oligonucleotides are included in the main text and Supplementary Material.

## Supplementary Material

gkac745_Supplemental_File

## References

[1] Lee M. , Wang X., Zhang S., Zhang Z., Lee E.Y.C. Regulation and modulation of human DNA polymerase delta activity and function. Genes (Basel). 2017; 8:190. 10.3390/genes8070190 PMC5541323 28737709

[2] Hedglin M. , Pandey B., Benkovic S.J. Stability of the human polymerase delta holoenzyme and its implications in lagging strand DNA synthesis. Proc. Natl. Acad. Sci. U.S.A. 2016; 113:E1777–E1786. 26976599 10.1073/pnas.1523653113 PMC4822616

[3] Hedglin M. , Kumar R., Benkovic S.J. Replication clamps and clamp loaders. Cold Spring Harb. Perspect. Biol. 2013; 5:a010165. 23545418 10.1101/cshperspect.a010165 PMC3683903

[4] Chen R. , Wold M.S. Replication protein A: single-stranded DNA’s first responder: dynamic DNA-interactions allow replication protein a to direct single-strand DNA intermediates into different pathways for synthesis or repair. Bioessays. 2014; 36:1156–1161. 25171654 10.1002/bies.201400107 PMC4629251

[5] Hedglin M. , Aitha M., Benkovic S.J. Monitoring the retention of human proliferating cell nuclear antigen at primer/template junctions by proteins that bind single-stranded DNA. Biochemistry. 2017; 56:3415–3421. 28590137 10.1021/acs.biochem.7b00386 PMC5662943

[6] Hedglin M. , Benkovic S.J. Replication protein a prohibits diffusion of the PCNA sliding clamp along single-stranded DNA. Biochemistry. 2017; 56:1824–1835. 28177605 10.1021/acs.biochem.6b01213 PMC5382571

[7] Hedglin M. , Benkovic S.J. Eukaryotic translesion DNA synthesis on the leading and lagging strands: unique detours around the same obstacle. Chem. Rev. 2017; 117:7857–7877. 28497687 10.1021/acs.chemrev.7b00046 PMC5662946

[8] Hedglin M. , Benkovic S.J. Regulation of rad6/rad18 activity during DNA damage tolerance. Annu. Rev. Biophys. 2015; 44:207–228. 26098514 10.1146/annurev-biophys-060414-033841 PMC5592839

[9] Fazlieva R. , Spittle C.S., Morrissey D., Hayashi H., Yan H., Matsumoto Y. Proofreading exonuclease activity of human DNA polymerase delta and its effects on lesion-bypass DNA synthesis. Nucleic Acids Res. 2009; 37:2854–2866. 19282447 10.1093/nar/gkp155 PMC2685094

[10] Narita T. , Tsurimoto T., Yamamoto J., Nishihara K., Ogawa K., Ohashi E., Evans T., Iwai S., Takeda S., Hirota K. Human replicative DNA polymerase delta can bypass T-T (6-4) ultraviolet photoproducts on template strands. Genes Cells. 2010; 15:1228–1239. 21070511 10.1111/j.1365-2443.2010.01457.x

[11] Markkanen E. , Castrec B., Villani G., Hubscher U. A switch between DNA polymerases delta and lambda promotes error-free bypass of 8-oxo-G lesions. Proc. Natl. Acad. Sci. U.S.A. 2012; 109:20401–20406. 23175785 10.1073/pnas.1211532109 PMC3528542

[12] Schmitt M.W. , Matsumoto Y., Loeb L.A. High fidelity and lesion bypass capability of human DNA polymerase delta. Biochimie. 2009; 91:1163–1172. 19540301 10.1016/j.biochi.2009.06.007 PMC2774493

[13] Meng X. , Zhou Y., Zhang S., Lee E.Y., Frick D.N., Lee M.Y. DNA damage alters DNA polymerase delta to a form that exhibits increased discrimination against modified template bases and mismatched primers. Nucleic Acids Res. 2009; 37:647–657. 19074196 10.1093/nar/gkn1000 PMC2632934

[14] Hirota K. , Tsuda M., Mohiuddin, Tsurimoto T., Cohen I.S., Livneh Z., Kobayashi K., Narita T., Nishihara K., Murai J. et al. In vivo evidence for translesion synthesis by the replicative DNA polymerase delta. Nucleic Acids Res. 2016; 44:7242–7250. 27185888 10.1093/nar/gkw439 PMC5009730

[15] Choi J.Y. , Chowdhury G., Zang H., Angel K.C., Vu C.C., Peterson L.A., Guengerich F.P. Translesion synthesis across O6-alkylguanine DNA adducts by recombinant human DNA polymerases. J. Biol. Chem. 2006; 281:38244–38256. 17050527 10.1074/jbc.M608369200

[16] Henricksen L.A. , Umbricht C.B., Wold M.S. Recombinant replication protein A: expression, complex formation, and functional characterization. J. Biol. Chem. 1994; 269:11121–11132. 8157639

[17] Hedglin M. , Perumal S.K., Hu Z., Benkovic S. Stepwise assembly of the human replicative polymerase holoenzyme. Elife. 2013; 2:e00278. 23577232 10.7554/eLife.00278 PMC3614016

[18] Li M. , Sengupta B., Benkovic S.J., Lee T.H., Hedglin M. PCNA monoubiquitination is regulated by diffusion of rad6/rad18 complexes along RPA filaments. Biochemistry. 2020; 59:4694–4702. 33242956 10.1021/acs.biochem.0c00849 PMC7887432

[19] Dolinnaya N.G. , Kubareva E.A., Romanova E.A., Trikin R.M., Oretskaya T.S. Thymidine glycol: the effect on DNA molecular structure and enzymatic processing. Biochimie. 2013; 95:134–147. 23000318 10.1016/j.biochi.2012.09.008

[20] Traut T.W. Physiological concentrations of purines and pyrimidines. Mol. Cell. Biochem. 1994; 140:1–22. 7877593 10.1007/BF00928361

[21] Hedglin M. , Pandey B., Benkovic S.J. Characterization of human translesion DNA synthesis across a UV-induced DNA lesion. Elife. 2016; 5:e19788. 27770570 10.7554/eLife.19788 PMC5123862

[22] Kim C. , Paulus B.F., Wold M.S. Interactions of human replication protein a with oligonucleotides. Biochemistry. 1994; 33:14197–14206. 7947831 10.1021/bi00251a031

[23] Kim C. , Snyder R.O., Wold M.S. Binding properties of replication protein a from human and yeast cells. Mol. Cell. Biol. 1992; 12:3050–3059. 1320195 10.1128/mcb.12.7.3050-3059.1992 PMC364519

[24] Kim C. , Wold M.S. Recombinant human replication protein a binds to polynucleotides with low cooperativity. Biochemistry. 1995; 34:2058–2064. 7849064 10.1021/bi00006a028

[25] Chilkova O. , Stenlund P., Isoz I., Stith C.M., Grabowski P., Lundstrom E.B., Burgers P.M., Johansson E. The eukaryotic leading and lagging strand DNA polymerases are loaded onto primer-ends via separate mechanisms but have comparable processivity in the presence of PCNA. Nucleic Acids Res. 2007; 35:6588–6597. 17905813 10.1093/nar/gkm741 PMC2095795

[26] Kokoska R.J. , McCulloch S.D., Kunkel T.A. The efficiency and specificity of apurinic/apyrimidinic site bypass by human DNA polymerase eta and sulfolobus solfataricus dpo4. J. Biol. Chem. 2003; 278:50537–50545. 14523013 10.1074/jbc.M308515200

[27] McCulloch S.D. , Kokoska R.J., Garg P., Burgers P.M., Kunkel T.A. The efficiency and fidelity of 8-oxo-guanine bypass by DNA polymerases delta and eta. Nucleic Acids Res. 2009; 37:2830–2840. 19282446 10.1093/nar/gkp103 PMC2685079

[28] Zhang S. , Zhou Y., Trusa S., Meng X., Lee E.Y., Lee M.Y. A novel DNA damage response: rapid degradation of the p12 subunit of dna polymerase delta. J. Biol. Chem. 2007; 282:15330–15340. 17317665 10.1074/jbc.M610356200

[29] Li H. , Xie B., Zhou Y., Rahmeh A., Trusa S., Zhang S., Gao Y., Lee E.Y., Lee M.Y. Functional roles of p12, the fourth subunit of human DNA polymerase delta. J. Biol. Chem. 2006; 281:14748–14755. 16510448 10.1074/jbc.M600322200

[30] Zhou Y. , Meng X., Zhang S., Lee E.Y., Lee M.Y. Characterization of human DNA polymerase delta and its subassemblies reconstituted by expression in the multibac system. PLoS One. 2012; 7:e39156. 22723953 10.1371/journal.pone.0039156 PMC3377666

[31] Rahmeh A.A. , Zhou Y., Xie B., Li H., Lee E.Y., Lee M.Y. Phosphorylation of the p68 subunit of pol delta acts as a molecular switch to regulate its interaction with PCNA. Biochemistry. 2012; 51:416–424. 22148433 10.1021/bi201638e

[32] Podust V.N. , Chang L.S., Ott R., Dianov G.L., Fanning E. Reconstitution of human DNA polymerase delta using recombinant baculoviruses: the p12 subunit potentiates DNA polymerizing activity of the four-subunit enzyme. J. Biol. Chem. 2002; 277:3894–3901. 11711545 10.1074/jbc.M109684200

[33] Masuda Y. , Suzuki M., Piao J., Gu Y., Tsurimoto T., Kamiya K. Dynamics of human replication factors in the elongation phase of DNA replication. Nucleic Acids Res. 2007; 35:6904–6916. 17932049 10.1093/nar/gkm822 PMC2175312

[34] Hu Z. , Perumal S.K., Yue H., Benkovic S.J. The human lagging strand DNA polymerase delta holoenzyme is distributive. J. Biol. Chem. 2012; 287:38442–38448. 22942285 10.1074/jbc.M112.404319 PMC3493889

[35] Markkanen E. Not breathing is not an option: how to deal with oxidative DNA damage. DNA Repair (Amst.). 2017; 59:82–105. 28963982 10.1016/j.dnarep.2017.09.007

[36] Cadet J. , Douki T., Ravanat J.L. Oxidatively generated damage to the guanine moiety of DNA: mechanistic aspects and formation in cells. Acc. Chem. Res. 2008; 41:1075–1083. 18666785 10.1021/ar700245e

[37] Shafirovich V. , Geacintov N.E. Removal of oxidatively generated DNA damage by overlapping repair pathways. Free Radic Biol Med. 2017; 107:53–61. 27818219 10.1016/j.freeradbiomed.2016.10.507 PMC5418118

[38] Kanvah S. , Joseph J., Schuster G.B., Barnett R.N., Cleveland C.L., Landman U. Oxidation of DNA: damage to nucleobases. Acc. Chem. Res. 2010; 43:280–287. 19938827 10.1021/ar900175a

[39] Hogg M. , Wallace S.S., Doublie S. Bumps in the road: how replicative DNA polymerases see DNA damage. Curr. Opin. Struct. Biol. 2005; 15:86–93. 15718138 10.1016/j.sbi.2005.01.014

[40] Zahn K.E. , Wallace S.S., Doublie S. DNA polymerases provide a canon of strategies for translesion synthesis past oxidatively generated lesions. Curr. Opin. Struct. Biol. 2011; 21:358–369. 21482102 10.1016/j.sbi.2011.03.008 PMC3112272

[41] Khare V. , Eckert K.A. The proofreading 3′→5′ exonuclease activity of DNA polymerases: a kinetic barrier to translesion DNA synthesis. Mutat. Res. 2002; 510:45–54. 12459442 10.1016/s0027-5107(02)00251-8

[42] Margison G.P. , Santibanez Koref M.F., Povey A.C. Mechanisms of carcinogenicity/chemotherapy by O6-methylguanine. Mutagenesis. 2002; 17:483–487. 12435845 10.1093/mutage/17.6.483

[43] Rioux K.L. , Delaney S. 1,N(6)-Ethenoadenine: from molecular to biological consequencesdagger. Chem. Res. Toxicol. 2020; 33:2688–2698. 33112144 10.1021/acs.chemrestox.0c00326

[44] Maga G. , Villani G., Crespan E., Wimmer U., Ferrari E., Bertocci B., Hubscher U. 8-oxo-guanine bypass by human DNA polymerases in the presence of auxiliary proteins. Nature. 2007; 447:606–608. 17507928 10.1038/nature05843

[45] Kusumoto R. , Masutani C., Iwai S., Hanaoka F. Translesion synthesis by human DNA polymerase eta across thymine glycol lesions. Biochemistry. 2002; 41:6090–6099. 11994004 10.1021/bi025549k

[46] Lancey C. , Tehseen M., Raducanu V.S., Rashid F., Merino N., Ragan T.J., Savva C.G., Zaher M.S., Shirbini A., Blanco F.J. et al. Structure of the processive human pol delta holoenzyme. Nat. Commun. 2020; 11:1109. 32111820 10.1038/s41467-020-14898-6 PMC7048817

[47] Swan M.K. , Johnson R.E., Prakash L., Prakash S., Aggarwal A.K. Structural basis of high-fidelity DNA synthesis by yeast DNA polymerase delta. Nat. Struct. Mol. Biol. 2009; 16:979–986. 19718023 10.1038/nsmb.1663 PMC3055789

[48] Guilliam T.A. , Yeeles J.T. The eukaryotic replisome tolerates leading-strand base damage by replicase switching. EMBO J. 2021; 40:e107037. 33555053 10.15252/embj.2020107037 PMC7917549

[49] Aller P. , Duclos S., Wallace S.S., Doublie S. A crystallographic study of the role of sequence context in thymine glycol bypass by a replicative DNA polymerase serendipitously sheds light on the exonuclease complex. J. Mol. Biol. 2011; 412:22–34. 21781974 10.1016/j.jmb.2011.07.007 PMC3167065

[50] Tolentino J.H. , Burke T.J., Mukhopadhyay S., McGregor W.G., Basu A.K. Inhibition of DNA replication fork progression and mutagenic potential of 1, N6-ethenoadenine and 8-oxoguanine in human cell extracts. Nucleic Acids Res. 2008; 36:1300–1308. 18184697 10.1093/nar/gkm1157 PMC2275085

[51] Monsen V.T. , Sundheim O., Aas P.A., Westbye M.P., Sousa M.M., Slupphaug G., Krokan H.E. Divergent ss-hairpins determine double-strand versus single-strand substrate recognition of human alkb-homologues 2 and 3. Nucleic Acids Res. 2010; 38:6447–6455. 20525795 10.1093/nar/gkq518 PMC2965238

[52] Falnes P.O. , Bjoras M., Aas P.A., Sundheim O., Seeberg E. Substrate specificities of bacterial and human AlkB proteins. Nucleic Acids Res. 2004; 32:3456–3461. 15229293 10.1093/nar/gkh655 PMC443531

[53] Chen B. , Liu H., Sun X., Yang C.G. Mechanistic insight into the recognition of single-stranded and double-stranded DNA substrates by ABH2 and ABH3. Mol. Biosyst. 2010; 6:2143–2149. 20714506 10.1039/c005148a

