# Supplementary material for: Tracking of progressing human DNA polymerase δ holoenzymes reveals distributions of DNA lesion bypass activities
Source: Nucleic Acids Res. 2022 Sep 15;50(17):9893–908. doi: 10.1093/nar/gkac745 (PMC9508823; doi:10.1093/nar/gkac745)
Supplement: gkac745_Supplemental_File [file gkac745_supplemental_file.docx]

**Supporting Information**

**TITLE:** Tracking of progressing human DNA polymerase δ holoenzymes reveal distributions of DNA lesion bypass activities

^1^Rachel L. Dannenberg^1^, Joseph A. Cardina^1,+^, Kara G. Pytko^1^, and Mark Hedglin^1,*^

^*^Corresponding author, to whom correspondence may be addressed: Email: [muh218@psu.edu](mailto:muh218@psu.edu)

^+^Supported by a Benkovic Award for Undergraduate Research

**Affiliations**:

^1^Department of Chemistry, The Pennsylvania State University, University Park, PA 16802

**Experimental Methods**

*FRET-based assay to monitor extent of RFC-catalyzed loading of PCNA.* All experiments were performed at room temperature (23 ± 2 °C) in 1X Replication Buffer (25 mm HEPES, pH 7.5, 10 mm Mg(OAc)_2_, 125 mm KOAc) supplemented with 1 mm DTT, and the final ionic strength was adjusted to physiological (200 mm) by the addition of appropriate amounts of KOAc. All measurements were done in 16.100F-Q-10/Z15 sub-micro fluorometer cells (Starna Cells) in a Horiba Scientific Duetta-Bio fluorescence/absorbance spectrometer. Excitation and emission slit widths are each set to 5 nm, unless indicated otherwise. Reaction solutions are excited at 514 nm and the fluorescence emission intensities (*I*) are simultaneously monitored at 563 nm (*I*_563_, Cy3 FRET donor fluorescence emission maximum) and 665 nm (*I*_665_, Cy5 FRET acceptor fluorescence emission maximum) over time, recording *I* every 0.17 s. For each time point, E_FRET_ is calculated where $E_{FRET}=\frac{I_{665}}{I_{665}+I_{563}}$. E_FRET_ values for complete loading of PCNA onto a P/T junction were calculated based on a published FRET-based assay(1-9). A solution containing 110 nM of a Bio-Cy3P/T DNA (**Figure S1**), 440 nM neutravidin and 1 mM ATP is pre-incubated with RPA (330 nM heterotrimer). Then, Cy5-PCNA (100 nM homotrimer) is added. Finally, RFC (100 nM heteropentamer) is added, the resultant solution is mixed via pipetting, and E_FRET_ is monitored beginning 10 s after the addition of RFC. Data is plotted as a function of time after RFC addition with time courses adjusted for the time between the addition of RFC and the recording of E_FRET_ (Δt = 10 s).

**

Supporting Figures**

**Figure S1**. DNA substrates utilized in this study. For all P/T DNA substrates, the sequences and lengths (29 bp) of the double strand DNA (dsDNA) regions are identical and in agreement with the requirements for assembly of a single PCNA ring onto DNA by RFC (1,4,7). The ssDNA regions adjacent to the 3’-end of the P/T junctions accommodate 1 RPA molecule (10-12). RPA prevents loaded PCNA from sliding off the ssDNA end of the substrate (4). When pre-bound to neutravidin, the biotin attached to the 5’ end of a primer strand prevents loaded PCNA from sliding off the dsDNA end of the substrate.

**
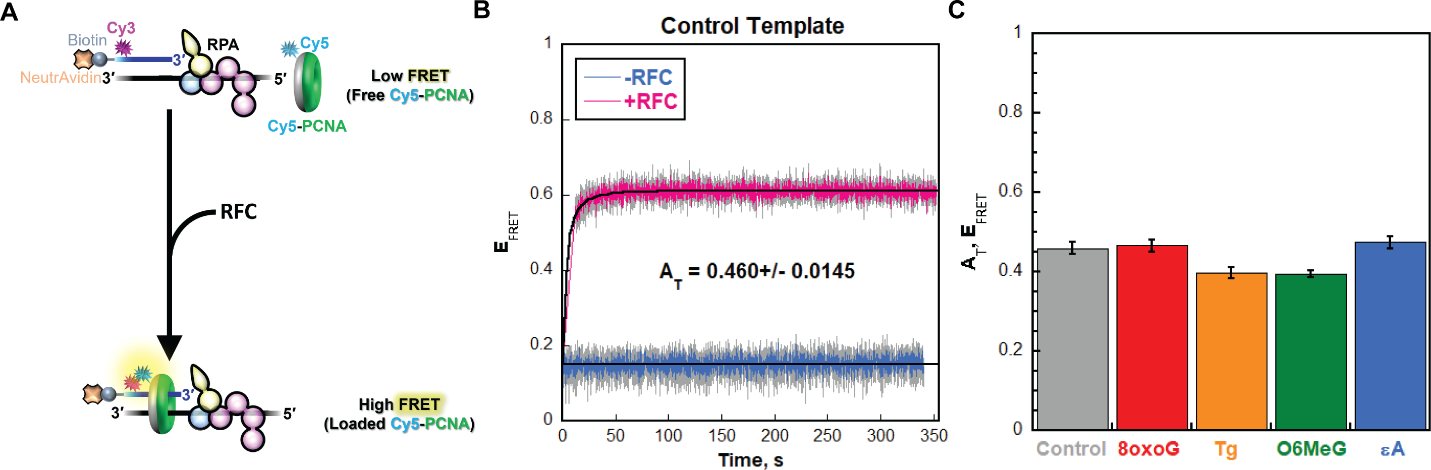
Figure S2.** RFC-catalyzed loading of PCNA onto DNA. (**A**) Schematic representation of the assay. The DNA substrates are P/T DNA substrates (**Figure S1**) in which the primer contains an internal Cy3 (FRET donor) 4 nt from its 5′ terminus. The “back face” of PCNA (shown in grey) is labeled with a Cy5 (FRET acceptor). Cy5-PCNA is loaded onto the DNA substrate by the human clamp loader, RFC, such that the Cy5 FRET donor on the “back face” of PCNA faces the Cy3 FRET donor near the 5′ terminus of the primer strand and the “front face” of PCNA (shown in green) is oriented towards the P/T junction where DNA synthesis emanates from. Cy5-PCNA is loaded by RFC in the presence of excess RPA and E_FRET_ is monitored over time. Under these conditions, all Cy5-PCNA is loaded onto a native (i.e., undamaged) BioCy3P/T DNA substrate (**Figure S1**) by RFC and stabilized by RPA and the biotin/neutravidin blocks that prevent diffusion of PCNA off of the DNA (1-9). (**B**) FRET data observed in the absence and presence of RFC for the BioCy3P/T-R DNA substrate (“control”). E_FRET_ is plotted as function of time after RFC is added. Each E_FRET_ trace is the average of at least three independent traces and the standard error is shown in grey. FRET is not observed when RFC is omitted and the signal remains constant. For these conditions, the traces are fit to a flat line. E_FRET_ increases with time only when RFC is included. For these conditions, the traces are fit to a double exponential rise where the sum of the amplitudes for the two phases (A_T_, shown) indicates the change in E_FRET_ observed when Cy5-PCNA is stably loaded onto the BioCy3P/T-R DNA substrate. Thus, A_T_ directly reports on the extent of stable assembly of PCNA onto DNA. (**C**) A_T_ data for the BioCy3P/T, BioCy3P/T-8oxoG, BioCy3P/T-Tg, BioCy3P/T-O6MeG, and BioCy3P/T-εA DNA substrates are shown in grey, red, orange, green, and blue, respectively. The A_T_ value observed for the control DNA substrate (i.e., native/undamaged) agrees with that observed previously (1-9), indicating that all Cy5-PCNA is loaded onto the P/T junction and stabilized. The A_T_ values observed for the DNA substrates containing a DNA lesion at least 9 nt downstream of the P/T junction are identical to that observed for control DNA substrate. This indicates that DNA lesions do not affect the stable assembly of PCNA onto a P/T junction upstream of the lesion.

**
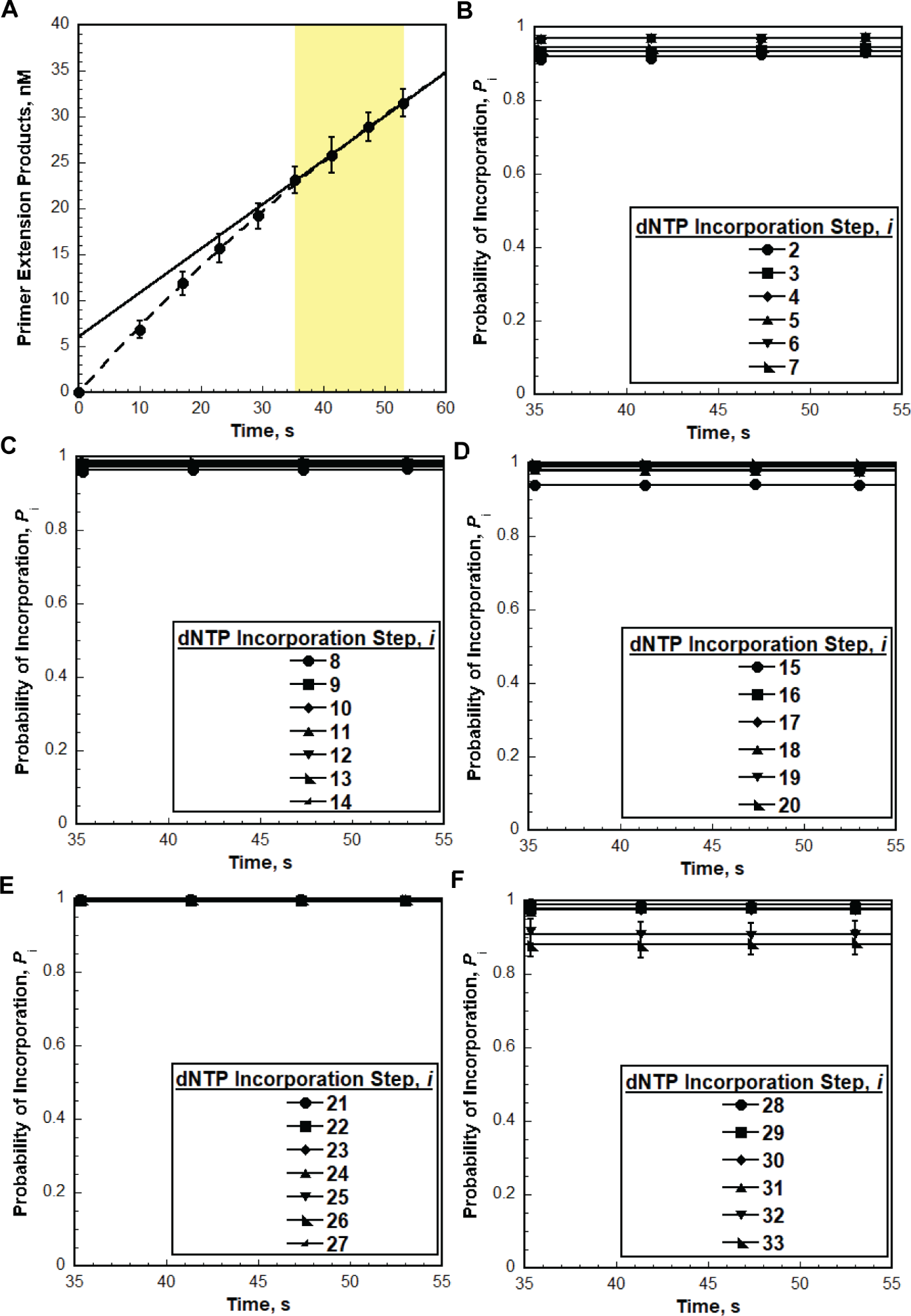
**

**Figure S3**. Monitoring primer extension by pol δ holoenzymes during a single binding encounter with a P/T DNA substrate. (**A**) Quantification of the (total) primer extension products for pol δ holoenzymes on the native (i.e., undamaged) P/T DNA substrate (BioCy3P/T, **Figure S1).** Data is identical to that displayed in **Figure 2C** in the main text. Each data point represents the average + S.E.M. of 3 independent experiments. Data is plotted as a function of time (after the addition of pol δ) and displays “burst” kinetics. Data points within the “linear” phase (highlighted in yellow) are fit to a liner regression where the Y-intercept (in nM) represents the amplitude of the “burst” phase and the slope represents the initial velocity (in nM/min) of the “linear” phase. (**B** - **F**) Processivity of pol δ holoenzymes at single nucleotide resolution. The probability of incorporation (*P*_i_) values for each dNTP incorporation step (*i*) beyond the first incorporation step (*i* = 2) are calculated for each time point within the “linear phase” of the reaction (indicated in panel **A**) for the experiments depicted in **Figure 2** and plotted as a function of time. The results for *i* = 2 to *i* = 7, *i* = 8 to *i* = 14, *i* = 15 to *i* = 20, *i* = 21 to *i* = 37, and *i* = 28 to *i* = 33 are depicted in panels **B**, **C**, **D**, **E**, and **F**, respectively, and each data point represents the average + S.E.M. of 3 independent experiments. For each dNTP incorporation step (*i*), *P*_i_ values remain constant within this incubation time. Identical behavior was observed for all P/T DNA substrates utilized in this study with all forms of pol δ.

|  |  | Pol δ | | Pol δ^Exo-^ | |
| --- | --- | --- | --- | --- | --- |
|  |  | Avg | StdErr | Avg | StdErr |
| Insertion Probability | G | 0.983 | 3.65E-04 | 0.964 | 1.75E-03 |
|  | 8oxoG | 0.842 | 6.34E-03 | 0.731 | 5.98E-03 |
| Insertion Efficiency (%) | | 85.7 | 0.646 | 75.8 | 0.635 |
| Extension Probability | G | 0.988 | 5.18E-04 | 0.967 | 1.97E-03 |
|  | 8oxoG | 0.432 | 0.0144 | 0.364 | 0.0143 |
| Extension Efficiency (%) | | 43.7 | 1.45 | 37.7 | 1.48 |
| Bypass Probability | G | 0.971 | 6.06E-04 | 0.932 | 3.56E-03 |
|  | 8oxoG | 0.364 | 0.0148 | 0.266 | 9.55E-03 |
| Bypass Efficiency (%) | | 37.5 | 1.52 | 28.6 | 1.030 |

**Table S1**. Efficiency of human pol δ holoenzymes replicating 8oxoG

|  |  | WT | | Exo- | |
| --- | --- | --- | --- | --- | --- |
|  |  | Avg | StdErr | Avg | StdErr |
| Insertion Probability | T | 0.979 | 9.73E-04 | 0.969 | 2.05E-03 |
|  | Tg | 0.775 | 6.56E-03 | 0.731 | 1.09E-02 |
| Insertion Efficiency (%) | | 79.1 | 0.674 | 75.4 | 1.133 |
| Extension Probability | T | 0.983 | 8.48E-04 | 0.974 | 1.96E-03 |
|  | Tg | 0.408 | 2.65E-02 | 0.129 | 1.12E-02 |
| Extension Efficiency (%) | | 41.5 | 2.696 | 13.3 | 1.150 |
| Bypass Probability | T | 0.963 | 1.78E-03 | 0.944 | 3.89E-03 |
|  | Tg | 0.316 | 0.0229 | 0.094 | 0.0068 |
| Bypass Efficiency (%) | | 32.9 | 2.38 | 10.0 | 0.72 |

**Table S2**: Efficiency of human pol δ holoenzymes replicating Tg

|  |  | WT | | Exo- | |
| --- | --- | --- | --- | --- | --- |
|  |  | Avg | StdErr | Avg | StdErr |
| Insertion Probability | G | 0.983 | 3.65E-04 | 0.964 | 1.75E-03 |
|  | O6MeG | 0.782 | 6.05E-03 | 0.646 | 7.97E-03 |
| Insertion Efficiency (%) | | 79.6 | 0.617 | 67.0 | 0.835 |
| Extension Probability | G | 0.988 | 5.18E-04 | 0.967 | 1.97E-03 |
|  | O6MeG | 0.854 | 2.44E-03 | 0.516 | 9.47E-03 |
| Extension Efficiency (%) | | 86.5 | 0.251 | 53.3 | 0.986 |
| Bypass Probability | G | 0.971 | 6.06E-04 | 0.932 | 3.56E-03 |
|  | O6MeG | 0.668 | 0.0060 | 0.334 | 0.0102 |
| Bypass Efficiency (%) | | 68.8 | 0.62 | 35.8 | 1.10 |

**Table S3**: Efficiency of human pol δ holoenzymes replicating O6MeG

|  |  | WT | | Exo- | |
| --- | --- | --- | --- | --- | --- |
|  |  | Avg | StdErr | Avg | StdErr |
| Insertion Probability | A | 0.983 | 8.48E-04 | 0.974 | 1.96E-03 |
|  | εA | 0.031 | 5.58E-03 | 0.042 | 3.01E-03 |
| Insertion Efficiency (%) | | 3.11 | 0.568 | 4.34 | 0.309 |
| Extension Probability | A | 0.972 | 1.15E-03 | 0.951 | 2.28E-03 |
|  | εA | 0.000 | 0.00E+00 | 0.000 | 0.00E+00 |
| Extension Efficiency (%) | | 0.0 |  | 0.0 |  |
| Bypass Probability | A | 0.955 | 1.91E-03 | 0.926 | 4.04E-03 |
|  | εA | 0.000 | 0.0000 | 0.000 | 0.0000 |
| Bypass Efficiency (%) | | 0.0 |  | 0.0 |  |

**Table S4**: Efficiency of human pol δ holoenzymes replicating εA.

**References**

1. Hedglin, M., Aitha, M. and Benkovic, S.J. (2017) Monitoring the Retention of Human Proliferating Cell Nuclear Antigen at Primer/Template Junctions by Proteins That Bind Single-Stranded DNA. *Biochemistry*, **56**, 3415-3421.

2. Hedglin, M., Aitha, M., Pedley, A. and Benkovic, S.J. (2019) Replication protein A dynamically regulates monoubiquitination of proliferating cell nuclear antigen. *J Biol Chem*, **294**, 5157-5168.

3. Hedglin, M. and Benkovic, S.J. (2017) Eukaryotic Translesion DNA Synthesis on the Leading and Lagging Strands: Unique Detours around the Same Obstacle. *Chem Rev*, **117**, 7857-7877.

4. Hedglin, M. and Benkovic, S.J. (2017) Replication Protein A Prohibits Diffusion of the PCNA Sliding Clamp along Single-Stranded DNA. *Biochemistry*, **56**, 1824-1835.

5. Hedglin, M., Pandey, B. and Benkovic, S.J. (2016) Stability of the human polymerase delta holoenzyme and its implications in lagging strand DNA synthesis. *Proc Natl Acad Sci U S A*, **113**, E1777-1786.

6. Hedglin, M., Pandey, B. and Benkovic, S.J. (2016) Characterization of human translesion DNA synthesis across a UV-induced DNA lesion. *Elife*, **5**, e19788, 19781 - 19718.

7. Hedglin, M., Perumal, S.K., Hu, Z. and Benkovic, S. (2013) Stepwise assembly of the human replicative polymerase holoenzyme. *Elife*, **2**, e00278, 00271 - 00220.

8. Li, M., Larsen, L. and Hedglin, M. (2020) Rad6/Rad18 Competes with DNA Polymerases eta and delta for PCNA Encircling DNA. *Biochemistry*.

9. Li, M., Sengupta, B., Benkovic, S.J., Lee, T.H. and Hedglin, M. (2020) PCNA Monoubiquitination Is Regulated by Diffusion of Rad6/Rad18 Complexes along RPA Filaments. *Biochemistry*.

10. Kim, C., Paulus, B.F. and Wold, M.S. (1994) Interactions of human replication protein A with oligonucleotides. *Biochemistry*, **33**, 14197-14206.

11. Kim, C., Snyder, R.O. and Wold, M.S. (1992) Binding properties of replication protein A from human and yeast cells. *Mol Cell Biol*, **12**, 3050-3059.

12. Kim, C. and Wold, M.S. (1995) Recombinant human replication protein A binds to polynucleotides with low cooperativity. *Biochemistry*, **34**, 2058-2064.
